# Distributed Fusion Filtering with Prediction Compensation for Multi-Sensor Systems Subject to DoS-Attack-Induced Packet Dropouts

**DOI:** 10.3390/s26175633

**Published:** 2026-09-04

**Authors:** Fengtao Hu, Jing Ma

**Affiliations:** 1 School of Mathematics and Statistics, Heilongjiang University, Harbin 150080, China; stephan_hu@163.com; 2 Heilongjiang Provincial Key Laboratory of the Theory and Computation of Complex Systems, Harbin 150080, China

**Keywords:** distributed fusion, denial-of-service attacks, prediction compensation, innovation analysis approach

## Abstract

This paper investigates the distributed fusion estimation problem for multi-sensor cyber-physical systems (CPSs), where the communication channels from local estimators to the fusion center are subject to random packet dropouts. Packet dropouts induced by either network congestion or intermittent denial-of-service (DoS) attacks are modeled as Bernoulli random variables. When a local estimate is lost, a prediction compensation strategy is adopted at the fusion center, where the missing data are replaced by their one-step predictors. By constructing an augmented state consisting of the original state, local prediction errors, and virtual measurements, the multi-sensor system is transformed into a stochastic system with random parameter matrices and one-step autocorrelated noises. Based on the transformed system, a distributed state fusion (DSF) filter is proposed via the innovation analysis method, whose filter gain depends on the successful-reception probabilities. The stability of the proposed DSF filter is analyzed, and a sufficient condition for the existence of a steady-state filter is obtained. The steady-state gain can be pre-computed offline, thereby reducing the online computational burden. Simulation results validate the effectiveness of the proposed algorithm.

## 1. Introduction

With the rapid development of CPSs, multi-sensor information fusion is critical in applications such as autonomous driving, Unmanned Aerial Vehicle (UAV) formation, smart grid monitoring, and industrial automation [1,2]. By combining complementary sensors like radar, LiDAR, cameras, IMU, and GPS, multi-sensor systems achieve better reliability and accuracy than single-sensor systems. However, the growing reliance on open communication networks exposes these systems to a key threat: DoS attacks [3]. In practice, such attacks degrade data availability by jamming channels or causing bursty packet delays and losses [4]. In a multi-sensor fusion scenario, even an intermittent DoS attack can block sensor data streams, resulting in incomplete, outdated, or irregularly sampled measurements at the fusion center. This can seriously degrade estimation performance and may even cause the filter to diverge. Therefore, designing a fusion filter that handles DoS attacks is a key issue in state estimation.

Various approaches have been proposed to model DoS attacks. For constant-rate attacks, ref. [5] uses a stochastic differential equation with Lévy jump noise to quantify their impact. In UAV confrontation scenarios, ref. [6] formulates the attack-defense interaction as a zero-sum game. For nonlinear CPSs with unknown dynamics, ref. [7] treats frequency-duration-constrained DoS attacks as intermittent channel blockages. In [8], energy-constrained attacks are modeled as multi-mode switching based on attack frequency and duration bounds. Unlike the models in [5,6,7,8], which account for adversarial intelligence and temporal correlations, another common approach in the literature relies on Bernoulli variables [9,10,11,12,13,14,15,16]. This model is mathematically equivalent to random packet losses caused by network congestion or poor channel conditions, and it does not capture the time-correlated or energy-limited features emphasized in [5,6,7,8]. In this paper, we adopt the Bernoulli-type dropout model for the distributed fusion filtering problem under DoS-induced packet dropouts.

Within this Bernoulli packet-dropout modeling framework, considerable studies have been made on filter design and state estimation. For systems suffering from random packet dropouts in both sensor-to-controller and controller-to-actuator channels, an optimal $H_{\infty }$ filter is proposed in [17] where two independent sets of Bernoulli random variables are introduced to describe packet loss phenomena separately. Similarly, focusing on the packet dropout problem with two sides from a sensor to an estimator and from a controller to an actuator, ref. [18] further considers bounded random transmission delays and obtains the optimal linear estimators including a filter, predictor and smoother. However, refs. [17,18] are only applicable to single-rate sampling systems. With the widespread adoption of multi-rate sampling systems, a least-squares linear estimator for multi-rate uncertain systems under DoS attacks is developed in [10]. The compensation strategy used for packet loss in [17] is the hold-input (HI) strategy, where the input is held at the most recent data that are received. In contrast, ref. [10] adopts both the HI and prediction compensation (PC) strategies. In the PC strategy, the lost measurement packet is compensated by its predictor. Compared with the HI strategy, the PC strategy exhibits stronger robustness against successive packet dropouts and achieves higher estimation accuracy, since the PC strategy utilizes all historical received data for compensation.

In contrast to the single-sensor systems in [10,17,18], multi-sensor systems are more popular [11,12,19,20,21,22]. Centralized and distributed fusion estimators are designed for systems under different sensors with different dropout rates in [19]. This framework is further extended to networked stochastic uncertain systems subject to transmission delays and packet dropouts [20]. Avoiding computing the second-order moment of the state which is required in [19,20], a matrix-weighted distributed fusion filter is designed in [21]. More recently, several additional practical engineering problems are also considered. Outlier-resistant sequential filtering fusion is addressed under DoS attacks with quantized measurements in [11]. The protocol-based distributed secure fusion estimation problem for networked systems with unknown bounded noise under quantization is considered in [12]. In the nonlinear domain, a fusion filter is developed where the local filtering gain matrix and the fusion weighting matrix are designed by solving convex optimization problems [22]. When the noise covariances are unknown but bounded, a robust dimensionality reduction fusion estimation is studied in [23]. Under the random parameter matrices, time-correlated additive noises, deception attacks and random packet dropouts, a centralized fusion linear estimator is developed by designing two strategies in [24]. To address the packet loss problem, the HI strategy is adopted in [12,19,20,22,23], the PC strategy is employed in [21] to compensate for lost data packets, and the zero-input (ZI) strategy, which sets the measurement to zero, is utilized in [11]. Among these three compensation strategies, the PC strategy achieves the best estimation performance.

In addition to single DoS attacks, hybrid attacks in which DoS attacks coexist with other attack types such as false data injection (FDI) or deception constitute another common scenario that has been investigated in some existing studies. From an application perspective, an adaptive distributed Kalman-like filter for power systems in [25] and event-triggered distributed fusion in [13] are studied. In addition, researchers also consider other system complexities: nonlinear hybrid attacks [14]; systems with quantized measurements [15]; systems involving random parameter matrices, correlated noises and quantized measurements [16] and multi-rate sampling [26]. The DoS attacks considered in the aforementioned literature are the losses of raw measurements. However, the transmission channels from estimators to the fusion center, as well as those between estimators, are also frequent targets of attackers, and the data lost over these channels are the state estimates [27,28,29,30]. Hybrid DoS and FDI attacks along with missing measurements are considered in [27], and a consensus estimator is proposed. For linear discrete systems, ref. [28] designs a distributed fusion filter with DoS compensation, but the process noise is uncorrelated with the measurement noise, and the measurement noises are mutually uncorrelated. Multi-sensor systems with missing data are considered in [29] and a consensus filter is proposed, in which not only the loss of raw measurements from local sensors is considered, but also the reception of local estimates from neighboring nodes is taken into account, and these local estimates are treated as measurement data. More recently, ref. [30] considers a large-scale clustered networks with a hierarchical event-triggered estimator. As shown in Table 1, none of the related works [13,14,15,16,21,25,26,27,28,29,30] simultaneously consider PC compensation for lost state estimates, correlated noises, and steady-state existence. This motivates the work presented in this paper.

Motivated by the above research gap, in this paper, we investigate a distributed fusion filtering problem for multi-sensor systems, where packet losses over the transmission channels from local estimators to the fusion center are modeled as Bernoulli random variables. Such packet dropouts can be induced by network congestion or DoS attacks. Consequently, the lost data packets are the local estimates rather than the raw measurements considered in [13,14,15,16,21,25,26]. When a local estimate is missing, the PC strategy is adopted; i.e., the missing data are replaced by their one-step predictors. Furthermore, the steady-state behavior of the proposed distributed state fusion (DSF) filter is rigorously analyzed, and a sufficient condition for the existence of the steady-state filter is established.

The main contributions of this paper are summarized as follows.

(1) This paper considers the multi-sensor system, where communication channels from local estimators to the fusion center are subject to Bernoulli packet dropouts. The system is transformed into an augmented system with random parameter matrices. The augmented state includes the original state, local prediction errors, and virtual measurements.
(2) For the augmented systems, a re-filtering-based DSF filter adopting the PC strategy is developed via the innovation analysis approach. Although the distributed weighted fusion (DWF) filter in [28] also considers DoS attacks on the channels from estimator to the fusion center and employs the PC strategy, the proposed DSF filter can handle correlated process and measurement noises. Furthermore, its steady-state filter gain can be pre-computed offline, which reduces the online computational cost.
(3) The proposed DSF filter is calculated using local one-step predictors that include historical raw measurements, whereas the fusion filter in [21] is based on the raw measurements that have missing data. Thus, the proposed DSF filter achieves superior fusion accuracy compared with that in [21] under the same packet reception probability. The steady-state existence is not discussed in [21].

It should be noted that the proposed DSF filter depends on the reception probabilities rather than random variables, which guarantees the existence of the steady-state filter. This may lead to a moderate accuracy loss compared with the DWF filter in [28] under low reception probabilities and independent noise, where the DWF filter achieves higher accuracy by using random variables. Under correlated noise conditions, DWF is not applicable, whereas the proposed DSF filter remains valid. This is further discussed in Section 6.

## 2. Problem Formulation

Consider the multi-sensor discrete linear stochastic system with *l* sensors:

(1) x(t+1)=Φx(t)+Γw(t),

(2) $$y_{i}\left(t\right)=H_{i}x\left(t\right)+v_{i}\left(t\right),i=1,2,\cdots ,l,$$

where $x\left(t\right)\in R^{n}$ is the state, $y_{i}\left(t\right)\in R^{m_{i}}$ is the raw measurement of the *i*-th sensor, *l* denotes the number of sensors, $w\left(t\right)\in R^{r}$ and $v_{i}\left(t\right)\in R^{m_{i}}$, i=1,2,⋯,l are the process and measurement noises of the *i*-th sensor, Φ, Γ and $H_{i}$, i=1,2,⋯,l are known constant matrices with appropriate dimensions.

**Assumption** **1.** *w(t) and $v_{i}\left(t\right)$, i=1,2,⋯,l are correlated white noises with the following statistical information:*

$$E\left\{\left[\begin{array}{c} w(t)\\ v_{i}\left(t\right) \end{array}\right]\left[\begin{array}{cc} w^{T}\left(k\right) & v_{j}^{T}\left(k\right) \end{array}\right]\right\}=\left[\begin{array}{cc} Q^{w} & S_{j}\\ S_{i}^{T} & Q_{ij}^{v} \end{array}\right]\delta_{t,k},$$

*where $Q_{ii}^{v}=Q_{i}^{v}$.*

**Assumption** **2.** *The initial state x(0) is uncorrelated with w(t) and $v_{i}\left(t\right)$, i=1,2,⋯,l and it satisfies*

$$E\left\{x\left(0\right)\right\}=x_{0},\;E\left\{\left[x\left(0\right)-x_{0}\right]{\left[x\left(0\right)-x_{0}\right]}^{T}\right\}=P_{0}.$$

First, we review the following Lemma 1, which will be used in the subsequent analysis.

**Lemma** **1** ([31])**.** *Given Assumptions 1 and 2, for the systems (1) and (2), the local one-step predictor ${\hat{x}}_{i}\left(t+1|t\right)$ derived from the raw measurements of the i-th sensor is expressed as*

(3) $${\hat{x}}_{i}\left(t+1|t\right)=\Psi_{i}\left(t\right){\hat{x}}_{i}\left(t|t-1\right)+L_{i}\left(t\right)y_{i}\left(t\right),$$

(4) $$L_{i}\left(t\right)=\left[\Phi P_{i}\left(t|t-1\right)H_{i}^{T}+\Gamma S_{i}\right]{\left[H_{i}P_{i}\left(t|t-1\right)H_{i}^{T}+Q_{i}^{v}\right]}^{-1},$$

(5) $$\begin{array}{cc} P_{i}\left(t+1|t\right) & =\Psi_{i}\left(t\right)P_{i}\left(t|t-1\right)\Psi_{i}^{T}\left(t\right)+\Gamma Q^{w}\Gamma^{T}-\Gamma S_{i}L_{i}^{T}\left(t\right)\\ \; & \;-L_{i}\left(t\right)S_{i}^{T}\Gamma^{T}+L_{i}\left(t\right)Q_{i}^{v}L_{i}^{T}\left(t\right), \end{array}$$

*where $\Psi_{i}\left(t\right)=\Phi -L_{i}\left(t\right)H_{i}$, the initial values are ${\hat{x}}_{i}\left(0|-1\right)=x_{0}$ and $P_{i}\left(0|-1\right)=P_{0}$.*

The local one-step predictor ${\hat{x}}_{i}\left(t+1|t\right)$ is designed based on the local measurements of the *i*-th sensor, i=1,2,⋯,l. The local one-step prediction is then transmitted to the fusion center. DoS attacks occur on the channels from the local estimators to the fusion center, and the packet loss phenomenon induced by DoS attacks is described by Bernoulli random variables $\gamma_{i}\left(t\right)$. $\gamma_{i}\left(t\right)=1$ means that the predictor ${\hat{x}}_{i}\left(t+1|t\right)$ and the corresponding gain matrices are successfully received by the fusion center, while $\gamma_{i}\left(t\right)=0$ indicates that ${\hat{x}}_{i}\left(t+1|t\right)$ is lost. Such packet dropouts can be caused by network congestion or, in particular, by DoS attacks modeled as Bernoulli variables. The successful data reception probabilities are $\mathrm{Prob}\left\{\gamma_{i}\left(t\right)=1\right\}=\alpha_{i}$ and the packet-dropout probabilities are $\mathrm{Prob}\left\{\gamma_{i}\left(t\right)=0\right\}=1-\alpha_{i}$, where $0\leq \alpha_{i}\leq 1$, i=1,2,⋯,l. The following stochastic independence properties of $\gamma_{i}\left(t\right)$, i=1,2,⋯,l, are assumed throughout this paper: (a) for each sensor, $\gamma_{i}\left(t\right)$ at different time instants are mutually independent; (b) $\gamma_{i}\left(t\right)$ and $\gamma_{j}\left(t\right)$ are mutually independent at the same time instant for i≠j; (c) the random variables $\gamma_{i}\left(t\right)$, i=1,2,⋯,l, are independent of other random variables in the considered system.

It should be pointed out that the Bernoulli model treats DoS-induced packet losses as independent random dropouts. Accordingly, it is not applicable to modeling intelligent and time-correlated attack scenarios.

**Remark** **1.** *The reception probabilities $\alpha_{i}$, i=1,2,⋯,l, can be obtained in practice via system identification. For example, we may analyze historical packet-loss records or employ the recursive extended least-squares algorithm for the online identification of packet reception rates using real-time received observation data (see [32] for the complete identification algorithm). Thus, it is assumed that the reception probabilities $\alpha_{i}$ are pre-identified and available. If the assumed $\alpha_{i}$ deviate from the true values, the covariance matrices obtained from Theorem 2 will no longer coincide with the actual filtering error covariances, and the performance degradation becomes more significant as the deviation increases. The sensitivity of the DSF filter to reception probability mismatches is examined via simulations in Section 6.*

**Figure 1:**
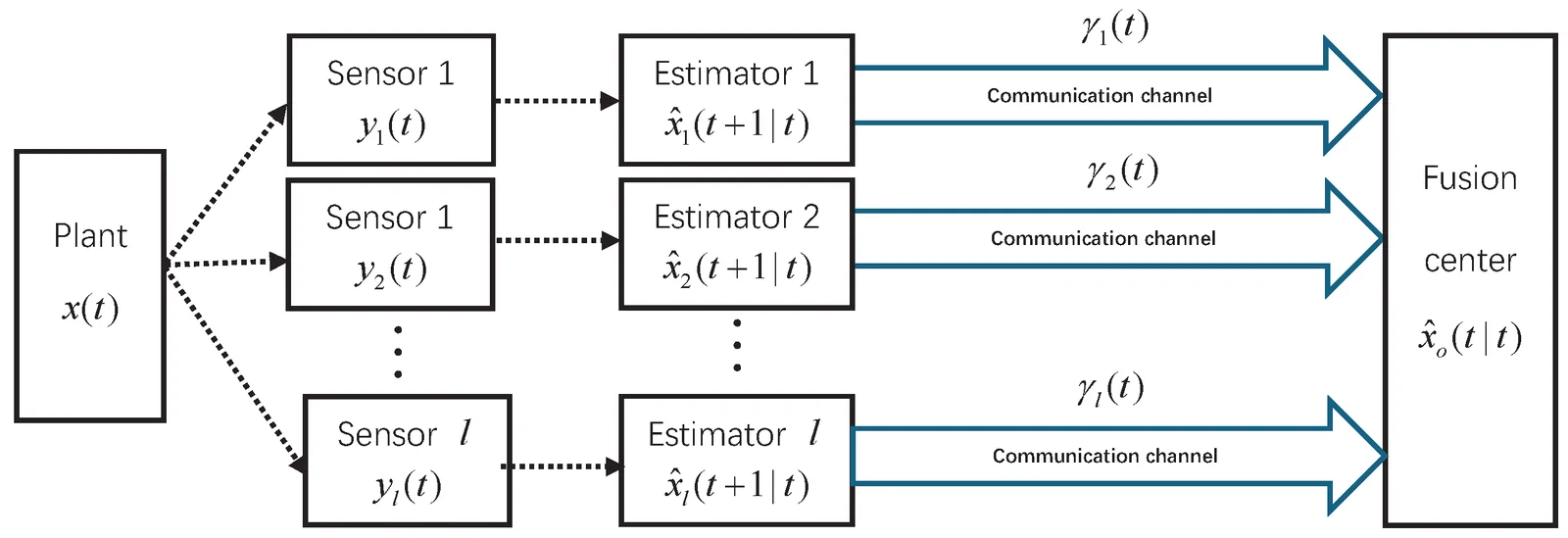
Communication and estimation setup of the multi-sensor system under DoS attacks.

For ease of understanding, the overall communication and estimation structure is illustrated in Figure 1. The aim of this paper is to design the DSF filter ${\hat{x}}_{o}\left(t|t\right)$ based on the received local one-step predictors ${\hat{x}}_{i}\left(t+1|t\right)$ with the gain matrices $L_{i}\left(t\right)$ in the fusion center.

In the next section, we will transform the system in the fusion center.

## 3. System Transformation

Let ${\bar{y}}_{i}\left(t\right)={\hat{x}}_{i}\left(t+1|t\right)=x\left(t+1\right)-{\tilde{x}}_{i}\left(t+1|t\right)$; then, the locally computed predictor ${\hat{x}}_{i}\left(t+1|t\right)$ can be regarded as a virtual measurement for the state x(t) (similar to [31]). The prediction error ${\tilde{x}}_{i}\left(t+1|t\right)$ is zero-mean under Assumptions 1 and 2, and its covariance is determined by the local Kalman filter. Therefore, the above relation satisfies the standard form of a measurement equation, namely, a linear function of the state together with zero-mean noise. In this formulation, the local prediction error $-{\tilde{x}}_{i}\left(t+1|t\right)$ acts as an equivalent ’measurement noise’. Thus, a new virtual measurement is constructed as

(6) $$z_{i}\left(t\right)=\gamma_{i}\left(t\right){\bar{y}}_{i}\left(t\right)+\left[1-\gamma_{i}\left(t\right)\right]{\hat{\bar{y}}}_{i}\left(t|t-1\right),i=1,2,\cdots ,l,$$

where ${\hat{\bar{y}}}_{i}\left(t|t-1\right)=\mathrm{proj}\left[{\bar{y}}_{i}\left(t\right)|z_{i}\left(1\right),z_{i}\left(2\right),\cdots ,z_{i}\left(t-1\right)\right]$, and proj[●|○] denotes the projection of ● onto the linear space L(○).

**Remark** **2.** *According to the projection theory [33], we have ${\hat{x}}_{i}\left(t+1|t\right)\in L\left(y_{i}\left(1\right),y_{i}\left(2\right),\cdots ,y_{i}\left(t\right)\right)$, i.e., ${\bar{y}}_{i}\left(t\right)\in L\left(y_{i}\left(1\right),y_{i}\left(2\right),\cdots ,y_{i}\left(t\right)\right),i=1,2,\cdots ,l$. From (6), we have $z_{i}\left(t\right)\in L\left({\bar{y}}_{i}\left(1\right),{\bar{y}}_{i}\left(2\right),\cdots ,{\bar{y}}_{i}\left(t\right)\right)$. Consequently, there is $L(z_{i}\left(1\right),z_{i}\left(2\right),\cdots ,z_{i}\left(t\right))$* ⊆ *$L({\bar{y}}_{i}\left(1\right),{\bar{y}}_{i}\left(2\right),\cdots ,{\bar{y}}_{i}\left(t\right))$* ⊆ *$L(y_{i}\left(1\right),y_{i}\left(2\right),\cdots ,y_{i}\left(t\right))$. From systems (1) and (2) and Assumption 1, we can obtain w(k)* ⊥ *$L(y_{i}\left(1\right),y_{i}\left(2\right),\cdots ,y_{i}\left(t\right))$, $v_{j}\left(k\right)$* ⊥ *$L(y_{i}\left(1\right),y_{i}\left(2\right),\cdots ,y_{i}\left(t\right))$, k>t, i,j=1,2,⋯,l. Hence, $w\left(k\right)⊥L(z_{i}\left(1\right),z_{i}\left(2\right),\cdots ,z_{i}\left(t\right))$ and $v_{j}\left(k\right)⊥L\left(z_{i}\left(1\right),z_{i}\left(2\right),\cdots ,z_{i}\left(t\right)\right)$, k>t, i,j=1,2,⋯,l.*

From (2) and (3) in Lemma 1, measurement input ${\bar{y}}_{i}\left(t\right)$ can be rewritten as

(7) $${\bar{y}}_{i}\left(t\right)=\Phi {\bar{y}}_{i}\left(t-1\right)+L_{i}\left(t\right)H_{i}{\tilde{x}}_{i}\left(t|t-1\right)+L_{i}\left(t\right)v_{i}\left(t\right).$$

When the DoS attacks occurs at time instant *t* and the t−1 time instant is free of DoS attacks, according to the projection theory [33], from (7), we have the one-step predictor of the measurement input ${\bar{y}}_{i}\left(t\right)$, i=1,2,⋯,l

(8) $$\begin{array}{cc} {\hat{\bar{y}}}_{i}\left(t|t-1\right) & =\mathrm{proj}[{\bar{y}}_{i}\left(t\right)|z_{i}\left(0\right),z_{i}\left(1\right),\cdots ,z_{i}\left(t-1\right)]\\ & =\Phi z_{i}\left(t-1\right)+\mathrm{proj}\left[L_{i}\left(t\right)H_{i}{\tilde{x}}_{i}\left(t|t-1\right)+L_{i}\left(t\right)v_{i}\left(t\right)|\right.\\ \; & \;\left.z_{i}\left(0\right),z_{i}\left(1\right),\cdots ,z_{i}\left(t-1\right)\right]. \end{array}$$

From the orthogonality of projection ${\tilde{x}}_{i}\left(t|t-1\right)⊥L\left(y_{i}\left(0\right),y_{i}\left(1\right),\cdots ,y_{i}\left(t-1\right)\right)$ and Remark 2, it can be derived that ${\tilde{x}}_{i}\left(t|t-1\right)⊥L\left(z_{i}\left(0\right),z_{i}\left(1\right),\cdots ,z_{i}\left(t-1\right)\right)$ and $v_{i}\left(t\right)$ ⊥ $L(z_{i}\left(0\right),z_{i}\left(1\right),\cdots ,z_{i}\left(t-1\right))$. Therefore, from (8), we can have

(9) $${\hat{\bar{y}}}_{i}\left(t|t-1\right)=\Phi z_{i}\left(t-1\right).$$

In fact, a compensator using all received local estimates can yield higher accuracy. However, it would introduce cross-correlations among local estimation errors, which would complicate the filter derivation. The compensator in (9) has the simple form $\Phi z_{i}\left(t-1\right)$ and relies only on the history of each local estimator. We adopt it as a design choice to keep the structure simple and the derivation feasible.

(9) is also applicable to consecutive packet dropouts. Without loss of generality, it is supposed that DoS attacks induce two successive packet loss events; i.e., packet loss occurs at both time instants t−1 and *t* but not at time instant t−2. From (6) and (9), we have

(10) $$z_{i}\left(t-1\right)={\hat{\bar{y}}}_{i}\left(t-1|t-2\right)=\Phi z_{i}\left(t-2\right),$$

thus, from (7), it can be obtained that

(11) $${\hat{\bar{y}}}_{i}\left(t|t-1\right)=\Phi^{2}z_{i}\left(t-2\right)=\Phi z_{i}\left(t-1\right).$$

By (1), (9) and the estimation error definition ${\tilde{x}}_{i}\left(t+1|t\right)=x\left(t+1\right)-{\hat{x}}_{i}\left(t+1|t\right)$, the virtual measurement $z_{i}\left(t\right)$ can be rewritten as

(12) $$z_{i}\left(t\right)=\gamma_{i}\left(t\right)\Phi x\left(t\right)-\gamma_{i}\left(t\right){\tilde{x}}_{i}\left(t+1|t\right)+\left[1-\gamma_{i}\left(t\right)\right]\Phi z_{i}\left(t-1\right)+\gamma_{i}\left(t\right)\Gamma w\left(t\right).$$

It follows from (1)–(3) that

(13) $${\tilde{x}}_{i}\left(t+1|t\right)=x\left(t+1\right)-{\hat{x}}_{i}\left(t+1|t\right)=\Psi_{i}\left(t\right){\tilde{x}}_{i}\left(t|t-1\right)+\Gamma w\left(t\right)-L_{i}\left(t\right)v_{i}\left(t\right).$$

From (1), (12) and (13), it can be obtained that x(t), $z_{i}\left(t-1\right)$ and ${\tilde{x}}_{i}\left(t+1|t\right)$ are temporally coupled. Therefore, these three components are augmented into a new state vector X(t) to avoid complicated derivations resulting from temporal coupling. Based on their respective recursive equations, the augmented state Equation (14) can then be constructed at the fusion center.

Let $X\left(t\right)={\left[x^{T}\left(t\right)\;{\tilde{x}}_{1}^{T}\left(t+1|t\right)\cdots {\tilde{x}}_{l}^{T}\left(t+1|t\right)\;z_{1}^{T}\left(t-1\right)\cdots z_{l}^{T}\left(t-1\right)\right]}^{T}$ be an augmented state and $z\left(t\right)={\left[z_{1}^{T}\left(t\right)\;z_{2}^{T}\left(t\right)\cdots z_{l}^{T}\left(t\right)\right]}^{T}$ be an augmented measurement. In the augmented state vector, x(t) is the original system state, ${\tilde{x}}_{i}\left(t+1|t\right)$ is the one-step prediction error of the *i*-th local estimator, and $z_{i}\left(t-1\right)$ is the virtual measurement. Using (1), (12) and (13), the systems (1) and (2) can be transformed at the fusion center into the following new system

(14) $$X\left(t+1\right)=\bar{\Phi }\left(t\right)X\left(t\right)+\bar{w}\left(t\right),$$

(15) z(t)=H(t)X(t)+D(t)w(t),

(16) $$\bar{w}\left(t\right)=\Gamma_{0}\left(t\right)w\left(t\right)+\Gamma_{1}w\left(t+1\right)+\Gamma_{2}\left(t+1\right)v\left(t+1\right),$$

where

$$\bar{\Phi }\left(t\right)=\left[\begin{array}{ccc} \Phi & 0 & 0\\ 0 & \Psi (t+1) & 0\\ \gamma \left(t\right)\Phi^{\left(1\right)} & -\gamma (t) & \left[I-\gamma \left(t\right)\right]\Phi^{\left(2\right)} \end{array}\right],$$

$$H\left(t\right)=\left[\begin{array}{ccc} \gamma \left(t\right)\Phi^{\left(1\right)} & -\gamma (t) & \left[I-\gamma \left(t\right)\right]\Phi^{\left(2\right)} \end{array}\right],\;D\left(t\right)=\gamma \left(t\right)\Gamma^{\left(1\right)},$$

$$\Gamma_{0}\left(t\right)=\left[\begin{array}{c} \Gamma \\ 0\\ \gamma \left(t\right)\Gamma^{\left(1\right)} \end{array}\right],\;\Gamma_{1}=\left[\begin{array}{c} 0\\ \Gamma^{\left(1\right)}\\ 0 \end{array}\right],\;\Gamma_{2}\left(t\right)=\left[\begin{array}{c} 0\\ -L^{a}\left(t\right)\\ 0 \end{array}\right],\;v\left(t\right)=\left[\begin{array}{c} v_{1}\left(t\right)\\ \vdots \\ v_{l}\left(t\right) \end{array}\right],$$

$$\Psi \left(t\right)=\mathrm{diag}\left(\Psi_{1}\left(t\right),\Psi_{2}\left(t\right),\cdots ,\Psi_{l}\left(t\right)\right),\;\gamma \left(t\right)=\mathrm{diag}\left(\gamma_{1}\left(t\right)I,\gamma_{2}\left(t\right)I,\cdots ,\gamma_{l}\left(t\right)I\right),$$

$$\Phi^{\left(1\right)}={\left[\begin{array}{ccc} \Phi^{T} & \cdots & \Phi^{T} \end{array}\right]}^{T},\;\Phi^{\left(2\right)}=\mathrm{diag}\left(\Phi ,\Phi ,\cdots ,\Phi \right),$$

$$\Gamma^{\left(1\right)}={\left[\begin{array}{ccc} \Gamma^{T} & \cdots & \Gamma^{T} \end{array}\right]}^{T},\;L^{a}\left(t\right)=\mathrm{diag}\left(L_{1}\left(t\right),L_{2}\left(t\right),\cdots ,L_{l}\left(t\right)\right),$$

0 is a zero matrix of appropriate dimensions, and I denotes the identity matrix of appropriate dimension. For clarity, the dimensions of the main quantities in (14)–(16) are summarized in Table 2.

**Figure 2:**
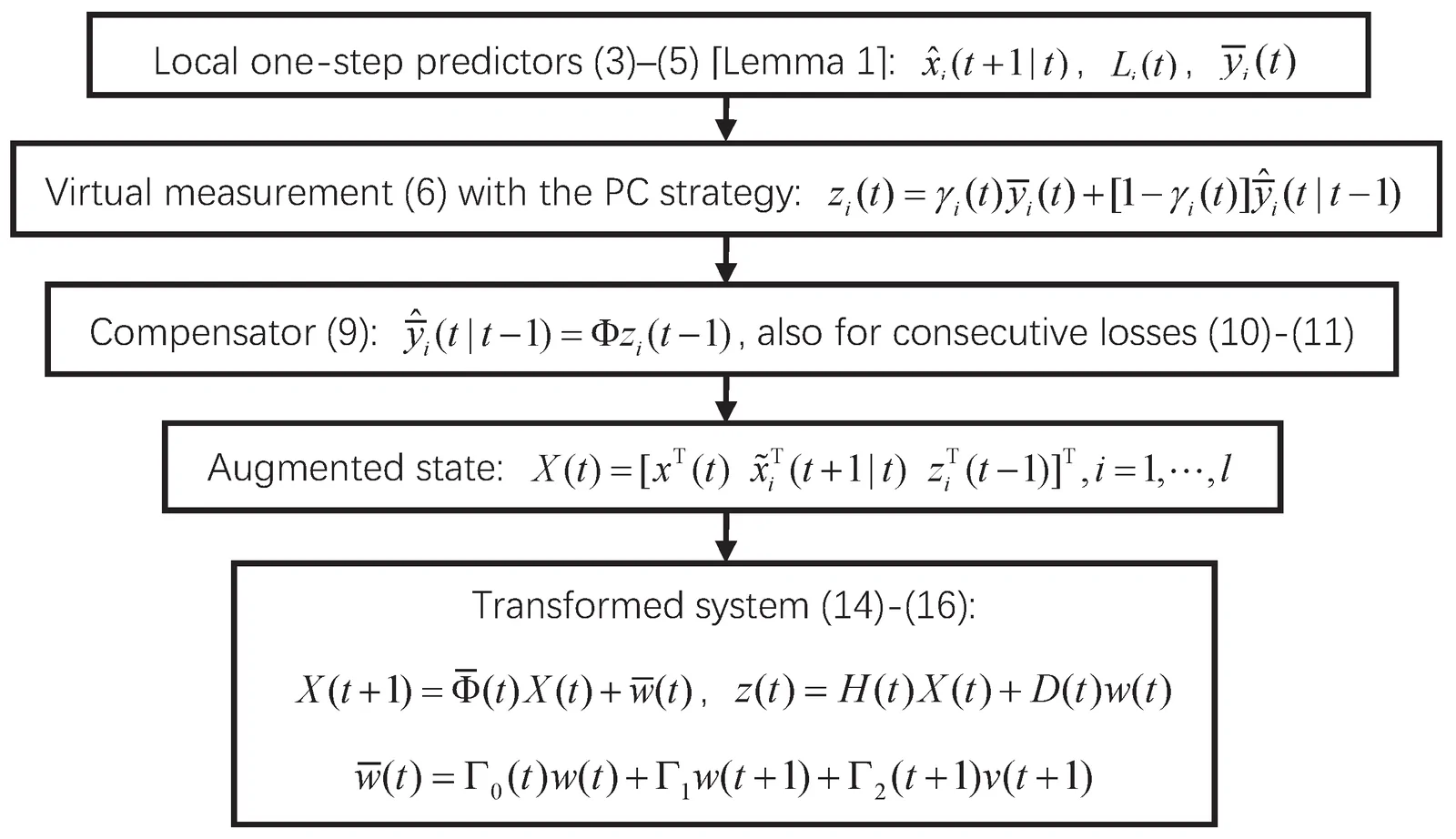
Schematic diagram illustrating the derivation flow of the system transformation procedure.

To facilitate understanding of the system transformation process, a simple schematic diagram of the derivation procedure is provided in Figure 2.

In the next section, a DSF filter is designed based on the transformed new system (14)–(16).

## 4. Distributed State Fusion Filter

In this section, the transformed new systems (14)–(16) are re-filtered via the innovation analysis approach in the fusion center, and the DSF filter is obtained. First of all, for ease of presentation, the following Remarks are given.

**Remark** **3.** (17) $$\begin{array}{cc} & {\left[\begin{array}{ccc} a_{1}I & \; & \\ \; & \ddots & \\ \; & \; & a_{k}I \end{array}\right]}_{r\times r}{\left(b_{ij}\right)}_{r\times s}{\left[\begin{array}{ccc} c_{1}I & \; & \\ \; & \ddots & \\ \; & \; & c_{l}I \end{array}\right]}_{s\times s}\\ \; & ={\left[\begin{array}{cccc} a_{1}c_{1}1_{m_{1}n_{1}} & a_{1}c_{2}1_{m_{1}n_{2}} & \cdots & a_{1}c_{l}1_{m_{1}n_{l}}\\ a_{2}c_{1}1_{m_{2}n_{1}} & a_{2}c_{2}1_{m_{2}n_{2}} & \cdots & a_{2}c_{l}1_{m_{2}n_{l}}\\ \vdots & \vdots & \ddots & \vdots \\ a_{k}c_{1}1_{m_{k}n_{1}} & a_{k}c_{2}1_{m_{k}n_{2}} & \cdots & a_{k}c_{l}1_{m_{k}n_{l}} \end{array}\right]}_{r\times s}⊙{\left(b_{ij}\right)}_{r\times s}, \end{array}$$

*where $1_{m_{k}n_{l}}$ denotes an $m_{k}\times n_{l}$ all-ones matrix.* ⊙ *is the Hadamard product with the definition $B⊙C={\left(b_{ij}\times c_{ij}\right)}_{r\times s}$, $B={\left(b_{ij}\right)}_{r\times s}$ and $C={\left(c_{ij}\right)}_{r\times s}$ are r×s matrices.*

**Remark** **4.** *Based on the probability distribution of the Bernoulli random variable $\gamma_{i}\left(t\right)$, we have that $E\left[\gamma_{i}\left(t\right)\right]=\alpha_{i}$, $E\left[\gamma_{i}^{2}\left(t\right)\right]=\alpha_{i}$, $E\left[\gamma_{i}\left(t\right)\gamma_{j}\left(t\right)\right]=\alpha_{i}\alpha_{j}$, i≠j, i,j=1,2,⋯,l.*

**Remark** **5.** *Regarding systems (14)–(16), the following results hold.*

$$\hat{\bar{\Phi }}\left(t\right)=E\left[\bar{\Phi }\left(t\right)\right]=\left[\begin{array}{ccc} \Phi & 0 & 0\\ 0 & \Psi (t+1) & 0\\ \alpha \Phi^{\left(1\right)} & -\alpha & \left[I-\alpha \right]\Phi^{\left(2\right)} \end{array}\right],\alpha =\mathrm{diag}\left(\alpha_{1}I,\alpha_{2}I,\cdots ,\alpha_{l}I\right),$$

$$\hat{H}=E\left[H\left(t\right)\right]=\left[\begin{array}{ccc} \alpha \Phi^{\left(1\right)} & -\alpha & \left[I-\alpha \right]\Phi^{\left(2\right)} \end{array}\right],\hat{D}=E\left[D\left(t\right)\right]=\alpha \Gamma^{\left(1\right)},{\hat{\Gamma }}_{0}=E\left[\Gamma_{0}\left(t\right)\right]=\left[\begin{array}{c} \Gamma \\ 0\\ \alpha \Gamma^{\left(1\right)} \end{array}\right].$$

*And*

$$\tilde{\bar{\Phi }}\left(t\right)=\bar{\Phi }\left(t\right)-\hat{\bar{\Phi }}\left(t\right)=\Lambda \left(t\right){\hat{\bar{\Phi }}}^{\left(1\right)},\;\tilde{H}\left(t\right)=H\left(t\right)-\hat{H}=\left[\gamma \left(t\right)-\alpha \right]{\hat{H}}^{\left(1\right)},$$

$$\tilde{D}\left(t\right)=D\left(t\right)-\hat{D}=\left[\gamma \left(t\right)-\alpha \right]\Gamma^{\left(1\right)},\;{\tilde{\Gamma }}_{0}\left(t\right)=\Gamma_{0}\left(t\right)-{\hat{\Gamma }}_{0}=\Lambda \left(t\right){\hat{\Gamma }}_{0}^{\left(1\right)},$$

*where*

$$\Lambda \left(t\right)=\left[\begin{array}{cc} 0 & 0\\ 0 & \gamma (t)-\alpha \end{array}\right],\;{\hat{\bar{\Phi }}}^{\left(1\right)}=\left[\begin{array}{ccc} 0 & 0 & 0\\ \Phi^{\left(1\right)} & -I & -\Phi^{\left(2\right)} \end{array}\right],$$

$${\hat{H}}^{\left(1\right)}=\left[\begin{array}{ccc} \Phi^{\left(1\right)} & -I & -\Phi^{\left(2\right)} \end{array}\right],\;{\hat{\Gamma }}_{0}^{\left(1\right)}=\left[\begin{array}{c} 0\\ \Gamma^{\left(1\right)} \end{array}\right].$$

*It can be observed that $E[\tilde{\bar{\Phi }}\left(t\right)]=0$, $E[\tilde{H}\left(t\right)]=0$, $E[\tilde{D}\left(t\right)]=0$, $E[{\tilde{\Gamma }}_{0}\left(t\right)]=0$.*

Under the Assumption 1, the noise statistics for systems (14)–(16) satisfy as follows.

**Lemma** **2.** *According to the Assumption 1, the system noises satisfy the following statistical characteristics.*

$$E\left[\bar{w}\left(t\right)\right]=0,\;E\left\{\left[\begin{array}{c} \bar{w}\left(t\right)\\ w(t) \end{array}\right]\left[\begin{array}{cc} {\bar{w}}^{T}\left(t\right) & w^{T}\left(t\right) \end{array}\right]\right\}=\left[\begin{array}{cc} Q^{\bar{w}}\left(t\right) & Q^{\bar{w}w}\\ Q^{w\bar{w}} & Q^{w} \end{array}\right],$$

(18) $$\begin{array}{cc} Q^{\bar{w}}\left(t\right) & =\left[\begin{array}{cc} 0 & 0\\ 0 & A \end{array}\right]⊙\left\{{\hat{\Gamma }}_{0}^{\left(1\right)}Q^{w}{\left[{\hat{\Gamma }}_{0}^{\left(1\right)}\right]}^{T}\right\}+{\hat{\Gamma }}_{0}Q^{w}{\hat{\Gamma }}_{0}^{T}+\Gamma_{1}Q^{w}\Gamma_{1}^{T}+\Gamma_{2}\left(t+1\right)Q^{v}\Gamma_{2}^{T}\left(t+1\right)\\ \; & \;+\Gamma_{1}S\Gamma_{2}^{T}\left(t+1\right)+\Gamma_{2}\left(t+1\right)S^{T}\Gamma_{1}^{T}, \end{array}$$

(19) $$Q^{\bar{w}w}={\hat{\Gamma }}_{0}Q^{w},$$

*where $Q^{w\bar{w}}={\left(Q^{\bar{w}w}\right)}^{T}$, $S=[S_{1}\;\cdots \;S_{l}]$, $Q^{v}={\left(Q_{ij}^{v}\right)}_{m\times m},i,j=1,2,\cdots ,l$ is a block matrix with $m=\sum_{i=1}^{l}m_{i}$, $A=\mathrm{diag}(\left(\alpha_{1}-\alpha_{1}^{2}\right)1_{nn},\cdots ,\left(\alpha_{l}-\alpha_{l}^{2}\right)1_{nn})$.*

**Proof.**  See the Appendix A. □

**Remark** **6.** *From (12), it can be seen that X(t)∈L(w(t),v(t),w(t−1),v(t−1),⋯,w(1),v(1),w(0)); under Assumption 1, we have w(j)⊥X(t) and v(j)⊥X(t), j>t. Since variable γ(t) is uncorrelated with all other variables, substituting (14) into $Q^{X\bar{w}}\left(t\right)=E\left[X\left(t\right){\bar{w}}^{T}\left(t\right)\right]$ under Assumption 1, there is*

$$Q^{X\bar{w}}\left(t\right)=\Gamma_{1}Q^{w}{\hat{\Gamma }}_{0}^{T}+\Gamma_{2}\left(t\right)S^{T}{\hat{\Gamma }}_{0}^{T}.$$

*Similarly, substituting (15) into $Q^{Xw}\left(t\right)=E\left[X\left(t\right)w^{T}\left(t\right)\right]$, there is*

$$Q^{Xw}\left(t\right)=\Gamma_{1}Q^{w}+\Gamma_{2}\left(t\right)S^{T},$$

*and $Q^{X\bar{w}}\left(t\right)=Q^{Xw}\left(t\right){\hat{\Gamma }}_{0}^{T}$.*

Before deriving the DSF filter, the following Theorem 1 is first presented.

**Theorem** **1.** *Under Assumptions 1 and 2 and Lemma 2, we can obtain the following second-order moment $q\left(t\right)=E[X\left(t\right)X^{T}\left(t\right)]$ of the state X(t) in system (14).*

(20) $$\begin{array}{cc} & q(t+1)\\ & =\hat{\bar{\Phi }}\left(t\right)q\left(t\right){\hat{\bar{\Phi }}}^{T}\left(t\right)+\left[\begin{array}{cc} 0 & 0\\ 0 & A \end{array}\right]⊙\left\{{\hat{\bar{\Phi }}}^{\left(1\right)}q\left(t\right){\left[{\hat{\bar{\Phi }}}^{\left(1\right)}\right]}^{T}\right\}+\hat{\bar{\Phi }}\left(t\right)Q^{X\bar{w}}\left(t\right)+Q^{\bar{w}X}\left(t\right){\hat{\bar{\Phi }}}^{T}\left(t\right)\\ \; & \;+Q^{\bar{w}}\left(t\right)+\left[\begin{array}{cc} 0 & 0\\ 0 & A \end{array}\right]⊙\left\{{\hat{\bar{\Phi }}}^{\left(1\right)}Q^{Xw}\left(t\right){\left[{\hat{\Gamma }}_{0}^{\left(1\right)}\right]}^{T}+{\hat{\Gamma }}_{0}^{\left(1\right)}Q^{wX}\left(t\right){\left[{\hat{\bar{\Phi }}}^{\left(1\right)}\right]}^{T}\right\}, \end{array}$$

*the initial value is $q\left(0\right)=\left[\begin{array}{ccc} x_{0}x_{0}^{T}+P_{0} & q^{x\tilde{x}}\left(0\right) & q^{x\hat{x}}\left(0\right)\\ q^{\tilde{x}x}\left(0\right) & q^{\tilde{x}\tilde{x}}\left(0\right) & q^{\tilde{x}\hat{x}}\left(0\right)\\ q^{\hat{x}x}\left(0\right) & q^{\hat{x}\tilde{x}}\left(0\right) & q^{\hat{x}\hat{x}}\left(0\right) \end{array}\right]$, where $q^{x\tilde{x}}\left(0\right)={\left(q^{x{\tilde{x}}_{i}}\right)}_{n\times nl}$, $q^{x\hat{x}}\left(0\right)={\left(q^{x{\hat{x}}_{i}}\right)}_{n\times nl}$, $q^{\tilde{x}\tilde{x}}\left(0\right)={\left(q^{{\tilde{x}}_{i}{\tilde{x}}_{j}}\right)}_{nl\times nl}$, $q^{\hat{x}\hat{x}}\left(0\right)={\left(q^{{\hat{x}}_{i}{\hat{x}}_{j}}\right)}_{nl\times nl}$, $q^{\tilde{x}\hat{x}}\left(0\right)=0$, $q^{x{\tilde{x}}_{i}}=P_{0}\Phi^{T}$, $q^{x{\hat{x}}_{i}}=x_{0}x_{0}^{T}$, $q^{{\tilde{x}}_{i}{\tilde{x}}_{j}}=\Phi P_{0}\Phi^{T}+\Gamma Q^{w}\Gamma^{T}$, $q^{{\hat{x}}_{i}{\hat{x}}_{j}}=x_{0}x_{0}^{T}$, i,j=1,2,⋯,l.*

**Proof.**  See Appendix B. □

In the following, a fusion filter is derived based on the noise statistical properties in Lemma 2, and the second-order moment q(t) is given in Theorem 1.

**Theorem** **2.** *Under Assumptions 1 and 2 for the system (14)–(16), the fusion filter $\hat{X}\left(t|t\right)$ and the one-step predictor are*

(21) $$\hat{X}\left(t|t\right)=\hat{X}\left(t|t-1\right)+K\left(t\right)\varepsilon \left(t\right),$$

(22) $$\hat{X}\left(t+1|t\right)=\hat{\bar{\Phi }}\left(t\right)\hat{X}\left(t|t-1\right)+L\left(t\right)\varepsilon \left(t\right).$$

*The innovation process ε(t) and the innovation covariance matrix $Q^{\varepsilon }\left(t\right)$ are*

(23) $$\varepsilon \left(t\right)=z\left(t\right)-\hat{H}\hat{X}\left(t|t-1\right),$$

(24) $$\begin{array}{cc} & Q^{\varepsilon }\left(t\right)=\hat{H}P\left(t|t-1\right){\hat{H}}^{T}+\hat{D}Q^{w}{\hat{D}}^{T}+\hat{H}Q^{Xw}\left(t\right){\hat{D}}^{T}+\hat{D}Q^{wX}\left(t\right){\hat{H}}^{T}+A\\ \; & \;⊙\left\{{\hat{H}}^{\left(1\right)}q\left(t\right){\left[{\hat{H}}^{\left(1\right)}\right]}^{T}\right.\left.+\Gamma^{\left(1\right)}Q^{w}{\left[\Gamma^{\left(1\right)}\right]}^{T}+{\hat{H}}^{\left(1\right)}Q^{Xw}\left(t\right){\left[\Gamma^{\left(1\right)}\right]}^{T}+\Gamma^{\left(1\right)}Q^{wX}\left(t\right){\left[{\hat{H}}^{\left(1\right)}\right]}^{T}\right\}, \end{array}$$

*where the innovation process ε(t) denotes the one-step prediction error of the measurement z(t), i.e., ε(t)=z(t)−proj[z(t)∣z(1),z(2),⋯,z(t−1)]. The filter gain matrix K(t) and the predictor gain matrix L(t) are*

(25) $$K\left(t\right)=\left[P\left(t|t-1\right){\hat{H}}^{T}+Q^{Xw}\left(t\right){\hat{D}}^{T}\right]{\left[Q^{\varepsilon }\left(t\right)\right]}^{-1},$$

(26) $$\begin{array}{cc} L(t)= & \left\{\left[\begin{array}{c} 0\\ A \end{array}\right]\;⊙\;\left\{\;{\hat{\bar{\Phi }}}^{\left(1\right)}q\left(t\right){\left[{\hat{H}}^{\left(1\right)}\right]}^{T}+{\hat{\bar{\Phi }}}^{\left(1\right)}Q^{Xw}\left(t\right){\left[\Gamma^{\left(1\right)}\right]}^{T}+{\hat{\Gamma }}_{0}^{\left(1\right)}Q^{wX}\left(t\right){\left[{\hat{H}}^{\left(1\right)}\right]}^{T}+{\hat{\Gamma }}_{0}^{\left(1\right)}Q^{w}\right.\right.\\ & \left.\left.\times {\left[\Gamma^{\left(1\right)}\right]}^{T}\right\}+\hat{\bar{\Phi }}\left(t\right)P\left(t|t-1\right){\hat{H}}^{T}+\hat{\bar{\Phi }}\left(t\right)Q^{Xw}\left(t\right){\hat{D}}^{T}+{\hat{\Gamma }}_{0}Q^{wX}\left(t\right){\hat{H}}^{T}+{\hat{\Gamma }}_{0}Q^{w}{\hat{D}}^{T}\right\}\\ \; & \times {\left[Q^{\varepsilon }\left(t\right)\right]}^{-1}. \end{array}$$

*The filtering error covariance matrix P(t|t) and the prediction error covariance matrix P(t+1|t) are*

(27) $$P\left(t|t\right)=P\left(t|t-1\right)-K\left(t\right)Q^{\varepsilon }\left(t\right)K^{T}\left(t\right),$$

(28) $$\begin{array}{cc} & P(t+1|t)\\ & =\hat{\bar{\Phi }}\left(t\right)P\left(t|t-1\right){\hat{\bar{\Phi }}}^{T}\left(t\right)+\hat{\bar{\Phi }}\left(t\right)Q^{X\bar{w}}\left(t\right)+Q^{\bar{w}X}\left(t\right){\hat{\bar{\Phi }}}^{T}\left(t\right)+Q^{\bar{w}}\left(t\right)-L\left(t\right)Q^{\varepsilon }\left(t\right)L^{T}\left(t\right)\\ \; & \;+\left[\begin{array}{cc} 0 & 0\\ 0 & A \end{array}\right]⊙\left\{{\hat{\bar{\Phi }}}^{\left(1\right)}q\left(t\right){\left[{\hat{\bar{\Phi }}}^{\left(1\right)}\right]}^{T}+{\hat{\bar{\Phi }}}^{\left(1\right)}Q^{Xw}\left(t\right){\left[{\hat{\Gamma }}_{0}^{\left(1\right)}\right]}^{T}+{\hat{\Gamma }}_{0}^{\left(1\right)}Q^{wX}\left(t\right){\left[{\hat{\bar{\Phi }}}^{\left(1\right)}\right]}^{T}\right\}. \end{array}$$

*The initial values are $\hat{X}\left(0|-1\right)={\left[\begin{array}{ccccccc} x_{0}^{T} & 0 & \cdots & 0 & x_{0}^{T} & \cdots & x_{0}^{T} \end{array}\right]}^{T}$ and $P\left(0|-1\right)=\left[\begin{array}{ccc} P_{0} & P^{x\tilde{x}}\left(0|-1\right) & 0\\ P^{\tilde{x}x}\left(0|-1\right) & P^{\tilde{x}\tilde{x}}\left(0|-1\right) & 0\\ 0 & 0 & 0 \end{array}\right]$, where $P^{x\tilde{x}}\left(0|-1\right)={\left(P^{x{\tilde{x}}_{i}}\right)}_{n\times nl}$, $P^{\tilde{x}\tilde{x}}\left(0|-1\right)={\left(P^{{\tilde{x}}_{i}{\tilde{x}}_{j}}\right)}_{nl\times nl}$, $P^{x{\tilde{x}}_{i}}=P_{0}\Phi^{T}$, $P^{{\tilde{x}}_{i}{\tilde{x}}_{j}}=\Phi P_{0}\Phi^{T}+\Gamma Q^{w}\Gamma^{T}$, $P^{\tilde{x}x}\left(0|-1\right)={\left[P^{x\tilde{x}}\left(0|-1\right)\right]}^{T}$, i,j=1,2,⋯,l.*

**Proof.**  See Appendix C. □

**Remark** **7.** *For systems (1) and (2) under Assumptions 1 and 2, the distributed fusion filter ${\hat{x}}_{o}\left(t|t\right)=\left[I_{n}\;0\right]\hat{X}\left(t|t\right)$ and the filtering error variance matrix $P_{o}\left(t|t\right)=\left[I_{n}\;0\right]P\left(t|t\right){\left[I_{n}\;0\right]}^{T}$ can be obtained.*

**Remark** **8.** *It should be pointed out that the augmented state vector X(t) is formed by augmenting x(t), ${\tilde{x}}_{i}\left(t+1|t\right)$ and $z_{i}\left(t-1\right)$, i=1,2,⋯,l. When the number of sensors l is large, the aggregate computational load across all local estimators grows, which may pose a non-negligible burden for real-time implementation. Moreover, transmitting both local predictor ${\hat{x}}_{i}\left(t+1|t\right)$ and gain matrices $L_{i}\left(t\right)$ additionally increases the communication burden. However, in this paper, the steady-state case is considered; i.e., $L_{i}\left(t\right)$ in Lemma 1, P(t+1|t), K(t) and L(t) in Theorem 2 converge to constant matrices. Consequently, the local gain matrices and the fusion gain matrices can be pre-computed offline, which significantly reduces the computational burden and avoids the need to transmit time-varying gain matrices $L_{i}\left(t\right)$ from the local estimators to the fusion center, thus further reducing the communication burden.*

To better demonstrate the implementing procedure of the proposed DSF filter algorithm, the computing steps are listed as follows:

Step 1 (Set the initial values). ${\hat{x}}_{i}\left(0|-1\right)=x_{0}$, $P_{i}\left(0|-1\right)=P_{0}$, i=1,2,⋯,l, $\hat{X}\left(0|-1\right)$, P(0|−1) and q(0).

Step 2 (Local predictor update). At each time *t*, for each sensor *i*, compute the gain $L_{i}\left(t\right)$ by (4), the local predictor ${\hat{x}}_{i}\left(t+1|t\right)$ by (3), and the local prediction error covariance $P_{i}\left(t+1|t\right)$ by (5). Transmit ${\hat{x}}_{i}\left(t+1|t\right)$ and $L_{i}\left(t\right)$ to the fusion center after calculation.

Step 3 (Packet reception/DoS realization). Observe the Bernoulli variables $\gamma_{i}\left(t\right)$: $\gamma_{i}\left(t\right)=1$ means that ${\hat{x}}_{i}\left(t+1|t\right)$ is received by the fusion center, while $\gamma_{i}\left(t\right)=0$ means that a DoS attack occurs and the packet is lost.

Step 4 (Prediction compensation). Compute the virtual measurement $z_{i}\left(t\right)$ by (6) and (9), and form $z\left(t\right)={\left[z_{1}^{T}\left(t\right)\;\cdots \;z_{l}^{T}\left(t\right)\right]}^{T}$.

Step 5 (Innovation computation). Compute the innovation ε(t) by (23).

Step 6 (Gain and covariance computation). Compute q(t+1) by (20), $Q^{\varepsilon }\left(t\right)$ by (24), and the gains K(t) by (25) and L(t) by (26).

Step 7 (Fusion update). Compute $\hat{X}\left(t|t\right)$ by (21), $\hat{X}\left(t+1|t\right)$ by (22), P(t|t) by (27), and P(t+1|t) by (28); output the fused estimate ${\hat{x}}_{o}\left(t|t\right)=\left[I_{n}\;0\right]\hat{X}\left(t|t\right)$.

Step 8 (Let *t* = t+1). Proceed to Step 2.

Each step above is implemented at each time *t*.

In the next section, the stability and steady-state behavior of the proposed DSF filter will be analyzed.

## 5. Stability Analysis and Steady-State Filter

Now, we will study the stability of the DSF filter obtained in Section 4.

First of all, to ensure the existence of a steady state for the local predictor, we make the following Assumption 3.

**Assumption** **3.** *$\left(\Phi ,H_{i}\right)$ is detectable, and $\left(\Phi -\Gamma S_{i}{\left(Q_{i}^{v}\right)}^{-1}H_{i},{\bar{R}}_{i}\right)$ is stabilizable, where ${\bar{R}}_{i}{\bar{R}}_{i}^{T}=\Gamma Q^{w}\Gamma^{T}-\Gamma S_{i}{\left(Q_{i}^{v}\right)}^{-1}S_{i}^{T}\Gamma^{T}$.*

From [33], we know that for systems (1) and (2) under Assumption 3, all local one-step predictors have steady states. Under the premise that the local predictor possesses a steady state, we present a sufficient condition for the convergence of the proposed DSF filter.

**Assumption** **4.** *$\rho (\hat{\bar{\Phi }}⊗\hat{\bar{\Phi }}+\sum_{i=1}^{l}\alpha_{i}\left(1-\alpha_{i}\right)I_{i}{\hat{\bar{\Phi }}}^{\left(1\right)}⊗{\hat{\bar{\Phi }}}^{\left(1\right)}I_{i})<1$, where* ⊗ *is the Kronecker product, $I_{i}=\mathrm{diag}\left(0,I,0\right)$ is a diagonal block matrix, the (l+1+i)-th diagonal block is an identity matrix of appropriate dimension, and ρ(•) is the spectrum radius of the matrix •.*

**Theorem** **3.** *For systems (14)–(16) under Assumptions 3 and 4, the solution q(t) to (20) with any initial value q(0)≥0 will converge to the unique positive semi-definite solution q to the following algebraic Lyapunov equation:*

(29) $$\begin{array}{cc} q & =\hat{\bar{\Phi }}q{\hat{\bar{\Phi }}}^{T}+\left[\begin{array}{cc} 0 & 0\\ 0 & A \end{array}\right]⊙\left\{{\hat{\bar{\Phi }}}^{\left(1\right)}q{\left[{\hat{\bar{\Phi }}}^{\left(1\right)}\right]}^{T}\right\}+\hat{\bar{\Phi }}Q^{X\bar{w}}+Q^{\bar{w}X}{\hat{\bar{\Phi }}}^{T}+Q^{\bar{w}}\\ \; & +\left[\begin{array}{cc} 0 & 0\\ 0 & A \end{array}\right]⊙\left\{{\hat{\bar{\Phi }}}^{\left(1\right)}Q^{Xw}{\left[{\hat{\Gamma }}_{0}^{\left(1\right)}\right]}^{T}+{\hat{\Gamma }}_{0}^{\left(1\right)}Q^{wX}{\left[{\hat{\bar{\Phi }}}^{\left(1\right)}\right]}^{T}\right\}. \end{array}$$

**Proof.**  See Appendix D. □

**Theorem** **4.** *For systems (14)–(16) under Theorem 3, if $\left(\hat{\bar{\Phi }},\hat{H}\right)$ is detectable, $\left(\hat{\bar{\Phi }}-\overset{˘}{S}{\overset{˘}{R}}^{-1}\hat{H},\bar{Q}\right)$ is stabilizable, where $\bar{Q}{\bar{Q}}^{T}=\overset{˘}{Q}-\overset{˘}{S}{\overset{˘}{R}}^{-1}{\overset{˘}{S}}^{T}$ and*

$$\begin{array}{cc} \overset{˘}{Q}= & \hat{\bar{\Phi }}Q^{Xw}{\hat{\Gamma }}_{0}^{T}+{\hat{\Gamma }}_{0}Q^{wX}{\hat{\bar{\Phi }}}^{T}+\Gamma_{1}Q^{w}\Gamma_{1}^{T}+\Gamma_{1}S\Gamma_{2}^{T}+\Gamma_{2}S^{T}\Gamma_{1}^{T}+\Gamma_{2}Q^{v}\Gamma_{2}^{T}\\ \; & +\left[\begin{array}{cc} 0 & 0\\ 0 & A \end{array}\right]⊙\left\{{\hat{\bar{\Phi }}}^{\left(1\right)}q{\left[{\hat{\bar{\Phi }}}^{\left(1\right)}\right]}^{T}+{\hat{\bar{\Phi }}}^{\left(1\right)}Q^{Xw}{\left[{\hat{\Gamma }}_{0}^{\left(1\right)}\right]}^{T}+{\hat{\Gamma }}_{0}^{\left(1\right)}Q^{w}{\left[{\hat{\Gamma }}_{0}^{\left(1\right)}\right]}^{T}+{\hat{\Gamma }}_{0}^{\left(1\right)}Q^{wX}{\left[{\hat{\bar{\Phi }}}^{\left(1\right)}\right]}^{T}\right\}, \end{array}$$

$$\begin{array}{cc} \overset{˘}{S}= & \left[\begin{array}{c} 0\\ A \end{array}\right]⊙\left\{{\hat{\bar{\Phi }}}^{\left(1\right)}q{\left[{\hat{H}}^{\left(1\right)}\right]}^{T}+{\hat{\bar{\Phi }}}^{\left(1\right)}Q^{Xw}{\left[\Gamma^{\left(1\right)}\right]}^{T}+{\hat{\Gamma }}_{0}^{\left(1\right)}Q^{wX}{\left[{\hat{H}}^{\left(1\right)}\right]}^{T}+{\hat{\Gamma }}_{0}^{\left(1\right)}Q^{w}{\left[\Gamma^{\left(1\right)}\right]}^{T}\right\}\\ \; & +\hat{\bar{\Phi }}Q^{Xw}{\hat{D}}^{T}+{\hat{\Gamma }}_{0}Q^{wX}{\hat{H}}^{T}+{\hat{\Gamma }}_{0}Q^{w}{\hat{D}}^{T}, \end{array}$$

$$\begin{array}{cc} \overset{˘}{R}= & \hat{H}Q^{Xw}{\hat{D}}^{T}+\hat{D}Q^{wX}{\hat{H}}^{T}+\hat{D}Q^{w}{\hat{D}}^{T}\\ \; & +A⊙\left\{{\hat{H}}^{\left(1\right)}q{\left[{\hat{H}}^{\left(1\right)}\right]}^{T}+{\hat{H}}^{\left(1\right)}Q^{Xw}{\left[\Gamma^{\left(1\right)}\right]}^{T}+\Gamma^{\left(1\right)}Q^{wX}{\left[{\hat{H}}^{\left(1\right)}\right]}^{T}+\Gamma^{\left(1\right)}Q^{w}{\left[\Gamma^{\left(1\right)}\right]}^{T}\right\}, \end{array}$$

*the solution P(t+1|t) to (28) with initial P(0|−1)≥0 will converge to the unique positive semi-definite solution Σ to the following algebraic Riccati equation:*

(30) $$\Sigma =\left(\hat{\bar{\Phi }}-L\hat{H}\right)\Sigma {\left(\hat{\bar{\Phi }}-L\hat{H}\right)}^{T}+\overset{˘}{Q}-L{\overset{˘}{S}}^{T}-\overset{˘}{S}L^{T}+L\overset{˘}{R}L^{T},$$

*Further, we have $L=\lim_{t\to \infty }L\left(t\right)$, $K=\lim_{t\to \infty }K\left(t\right)$ and $\Sigma =\lim_{t\to \infty }P\left(t|t-1\right)$.*

*Furthermore, the steady-state fusion predictor*

(31) $${\hat{X}}^{s}\left(t+1|t\right)=\left(\hat{\bar{\Phi }}-L\hat{H}\right){\hat{X}}^{s}\left(t|t-1\right)+Lz\left(t\right)$$

*is asymptotically stable.*

**Proof.**  See Appendix E. □

**Remark** **9.** *Compared with the real-time DSF filter computed by Steps 1–8 in the previous section, the steady-state DSF filter only requires Steps 1–5, 7 with the following replacements, where Steps 3, 4 and 5 remain unchanged.*

(1) *In Step 1, only ${\hat{X}}^{s}\left(0|-1\right)$ needs to be initialized and its value can be chosen arbitrarily, since the steady-state fusion predictor (31) is asymptotically stable.*
(2) *In Step 2, the time-varying gain $L_{i}\left(t\right)$ is replaced by its constant steady-state value $L_{i}$; i.e., only the predictor ${\hat{x}}_{i}\left(t+1|t\right)$ needs to be transmitted to the fusion center.*
(3) *Step 6 is omitted, because q(t), $Q^{\varepsilon }\left(t\right)$, K(t) and L(t) converge to the constant matrices q, $Q^{\varepsilon }$, K and L, which can be pre-computed offline according to Theorems 3 and 4.*
(4) *In Step 7, the fusion filter and predictor are updated by the steady-state filter ${\hat{X}}^{s}\left(t|t\right)={\hat{X}}^{s}\left(t|t-1\right)+K\varepsilon \left(t\right)$ and the steady-state predictor (31), where the innovation ε(t) is computed by (23) with $\hat{X}\left(t|t-1\right)$ replaced by ${\hat{X}}^{s}\left(t|t-1\right)$.*

## 6. Simulation Examples

### 6.1. Example 1

In this example, two cases are considered to compare the estimation accuracy: Case 1 with correlated noise and Case 2 with independent noise.

Consider a tracking system with three sensors, which is described as follows:

(32) $$x\left(t+1\right)=\left[\begin{array}{cc} 1 & T\\ 0 & 1 \end{array}\right]x\left(t\right)+\left[\begin{array}{c} T^{2}/2\\ T \end{array}\right]w\left(t\right),$$

(33) $$y_{i}\left(t\right)=H_{i}x\left(t\right)+v_{i}\left(t\right),\;i=1,2,3.$$

The sampling interval is T=0.25. The zero-mean white noise w(t) with variance $Q^{w}=1$ correlates with the measurement noises $v_{i}\left(t\right)$ via $v_{i}\left(t\right)=c_{i}w\left(t\right)+\eta_{i}\left(t\right)$, where $c_{i}$ represents the noise correlation coefficients. $\eta_{i}\left(t\right)$, i=1,2,3 are uncorrelated white noises with zero mean with the covariance matrices $Q^{\eta_{1}}=1$, $Q^{\eta_{2}}=2$, $Q^{\eta_{3}}=3$. The measurement matrices are $H_{1}=\left[\begin{array}{cc} 0.5 & 1 \end{array}\right]$, $H_{2}=\left[\begin{array}{cc} 1 & 1 \end{array}\right]$, $H_{3}=\left[\begin{array}{cc} 1 & 0.5 \end{array}\right]$. The packet reception probabilities of three sensors are set to $\alpha_{1}=0.9$, $\alpha_{2}=0.85$ and $\alpha_{3}=0.8$, respectively. The initial values are $x_{0}={\left[0\;0\right]}^{T}$ and $P_{0}=0.1I_{2}$, where $I_{2}$ denotes the 2×2 identity matrix.

Case 1 (correlated noise case): The noise correlation coefficients are set to $c_{1}=0.2$, $c_{2}=0.4$, and $c_{3}=0.6$.

**Figure 3:**
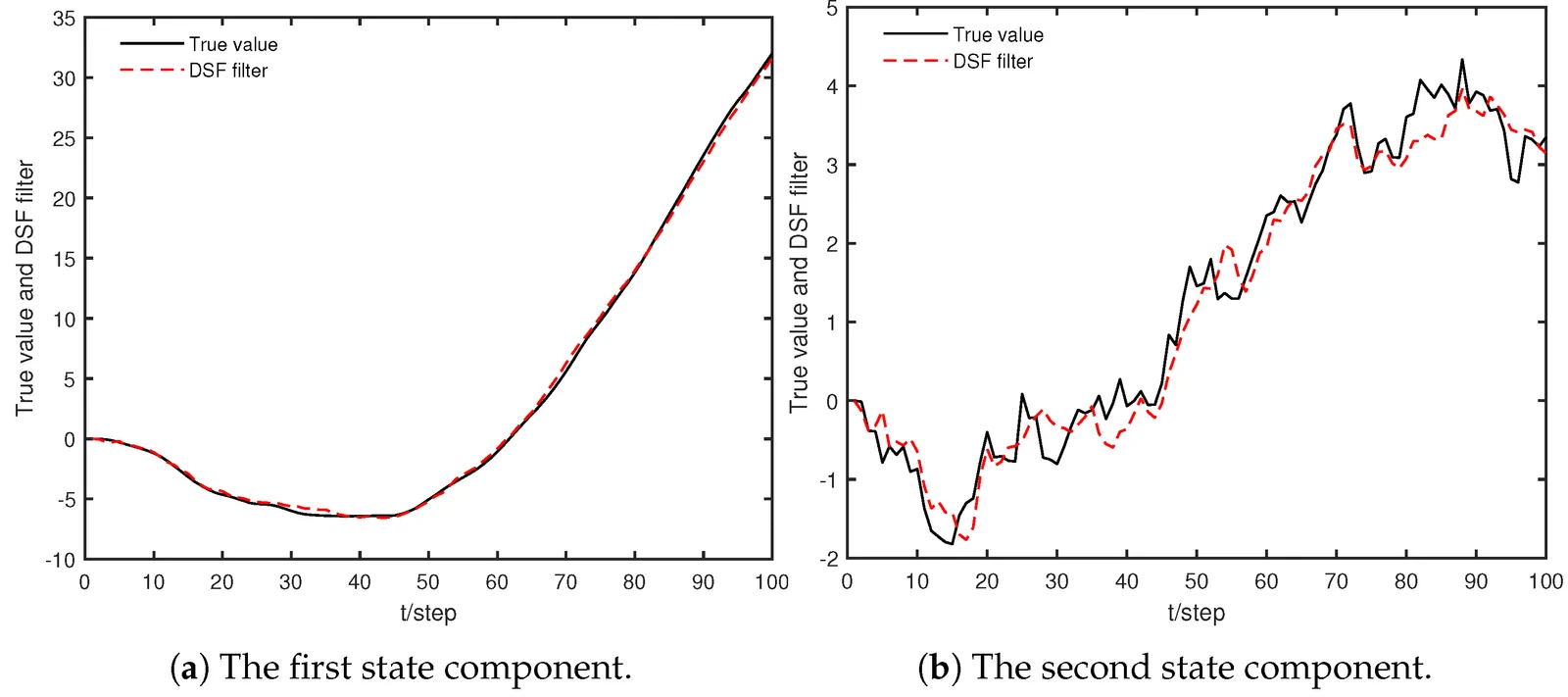
The tracking performance of DSF filter.

Figure 3 shows the tracking performance of the proposed fusion filter: (a) is the first state component and (b) is the second state component. It can be seen that the proposed DSF filter has the effective performance.

**Figure 4:**
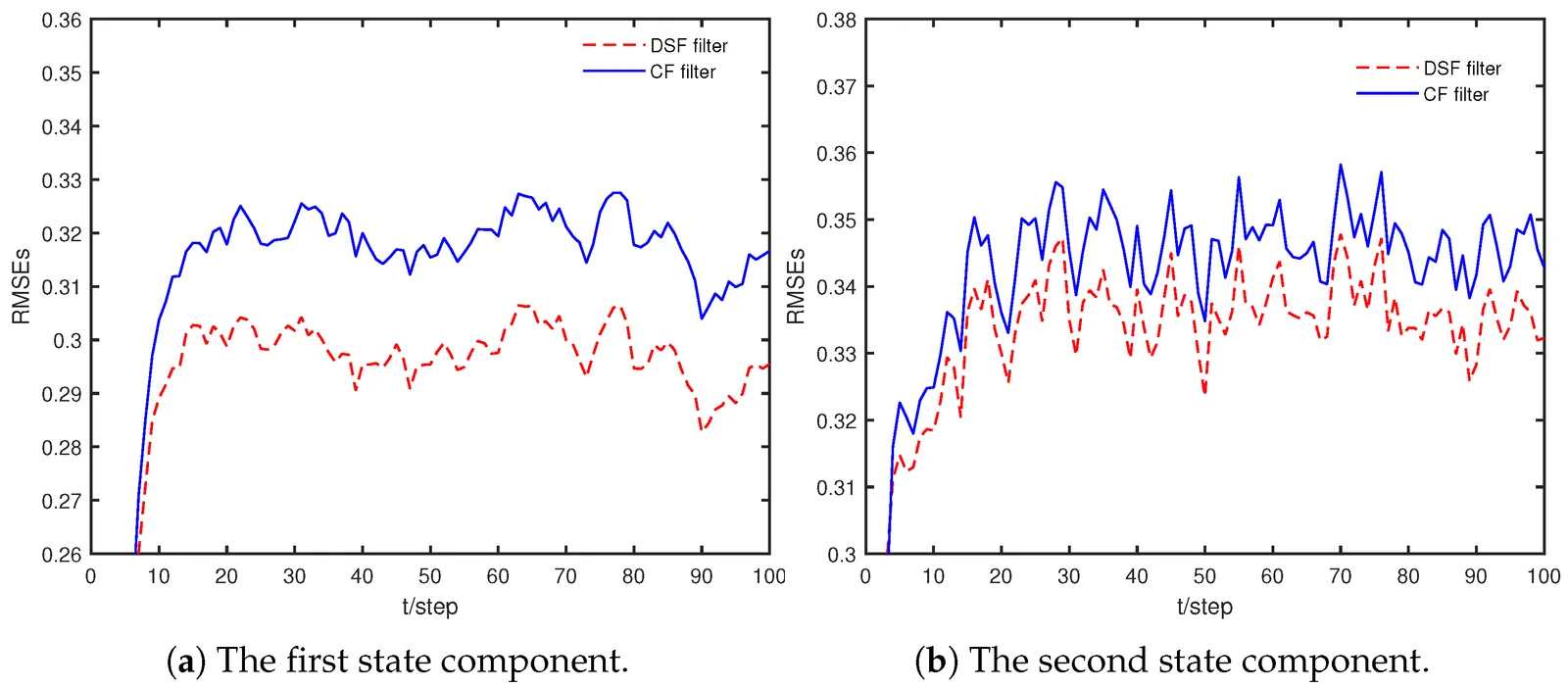
RMSEs of DSF filter and CF filter in [21].

Figure 4 shows the Root Mean Square Error based on 2000 Monte Carlo trials (RMSE). The RMSE for the *i*-th state component is defined as ${\mathrm{RMSE}}_{i}\left(t\right)=\sqrt{\frac{1}{M}\sum_{k=1}^{M}{\left(x_{i}^{k}\left(t\right)-{\hat{x}}_{i}^{k}\left(t|t\right)\right)}^{2}},$ where *M* is the number of Monte Carlo runs, $x_{i}^{k}\left(t\right)$ is the true value of the *i*-th state component in the *k*-th run, and ${\hat{x}}_{i}^{k}\left(t|t\right)$ is the corresponding filtered estimate. It can be observed that the RMSE values of DSF filter are lower than those of CF filter, which verifies the superior estimation accuracy of the proposed DSF filter.

The averaged steady-state RMSE ($ARMSE=\frac{1}{N-t_{0}+1}\sum_{t=t_{0}}^{N}\mathrm{RMSE}\left(t\right)$) computes from the 20th step to the 100th step, i.e., N=100, $t_{0}=20$. The ARMSE in all simulations is calculated using the definition given above. And the improvement is calculated as $Improvement=\frac{ARMSE_{\mathrm{ref}}-ARMSE_{\mathrm{DSF}}}{ARMSE_{\mathrm{ref}}}\times 100\%$, $ARMSE_{\mathrm{DSF}}$ denotes the ARMSE of the DSF filter, and $ARMSE_{\mathrm{ref}}$ denotes the ARMSE of the compared filter. As shown in Table 3, the ARMSEs of the proposed DSF filter are lower than those of the CF filter in [21] for both state components, with improvements of 7.02% and 2.81%, respectively, which further shows that the proposed filter achieves superior estimation accuracy.

**Figure 5:**
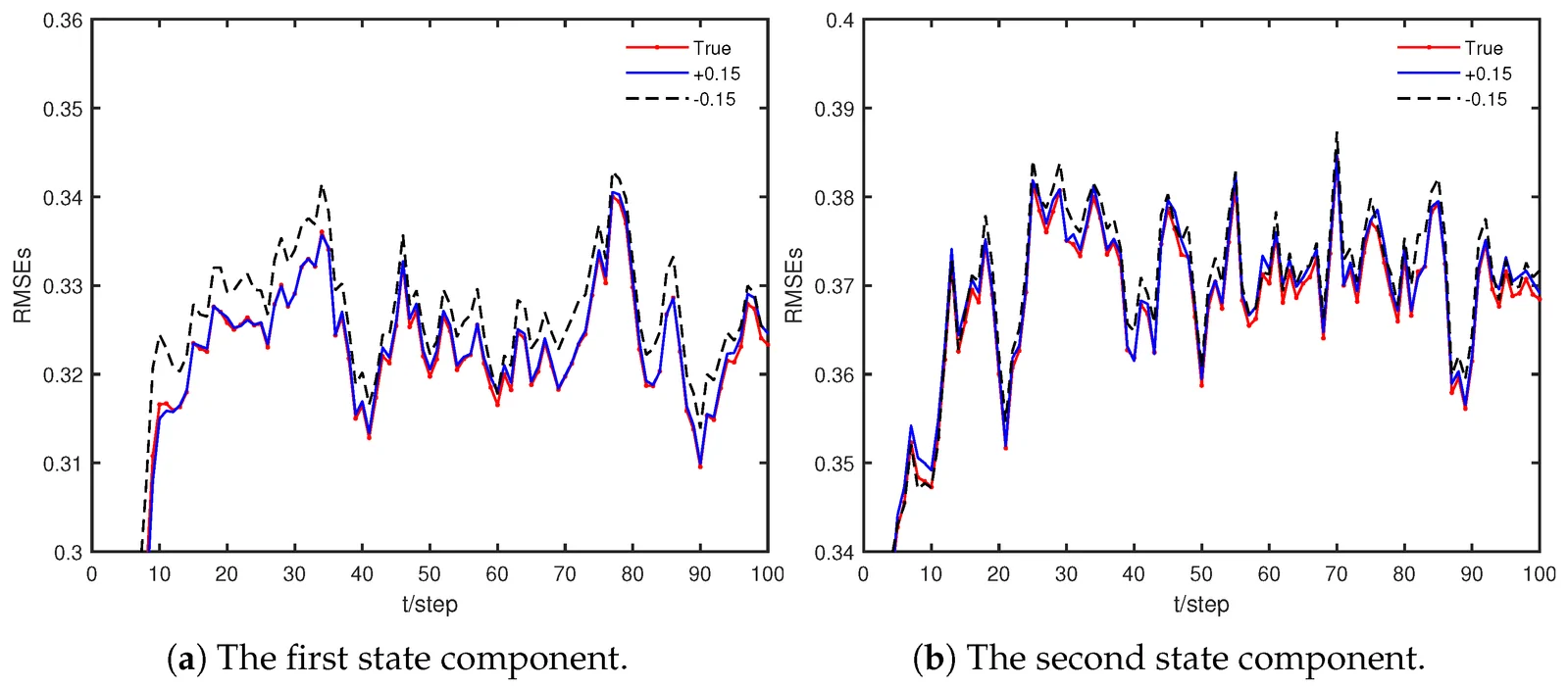
Sensitivity of the DSF filter to the reception probability mismatch.

We evaluate the sensitivity of the proposed filter to reception probabilities by setting the true probabilities to $\alpha_{1}=0.5$, $\alpha_{2}=0.6$, $\alpha_{3}=0.7$, and considering two mismatched cases with ($\alpha_{i}^{\prime }=\alpha_{i}\pm 0.15$). The variation rates of the reception probability and the ARMSE are computed as $Variation_{\alpha }=\frac{|\alpha_{i}-\alpha_{i}^{\prime }|}{\alpha_{i}}\times 100\%$ and $Variation_{ARMSE}=\frac{|ARMSE-ARMSE^{\prime }|}{ARMSE}\times 100\%$, where ARMSE and ARMSE’ correspond to the correct and mismatched cases, respectively. As shown in Table 4, when the assumed reception probabilities deviate from the true values by 0.15 (a relative change of 21.43% to 30%), the ARMSEs of the two state components change by no more than 1.05%. Figure 5 shows that the RMSE curves under these mismatches remain close to the correct-probability case. Figure 5 and Table 4 show that the DSF filter performance degrades only slightly under the considered mismatched cases.

Case 2 (uncorrelated noise case): In this case, the process noise w(t) is uncorrelated with the measurement noises $v_{i}\left(t\right)$, i=1,2,3, i.e., $c_{1}=c_{2}=c_{3}=0$. This setting is used to ensure a fair comparison with the DWF filter in [28], since the DWF filter is derived under the assumption of independent noises.

**Figure 6:**
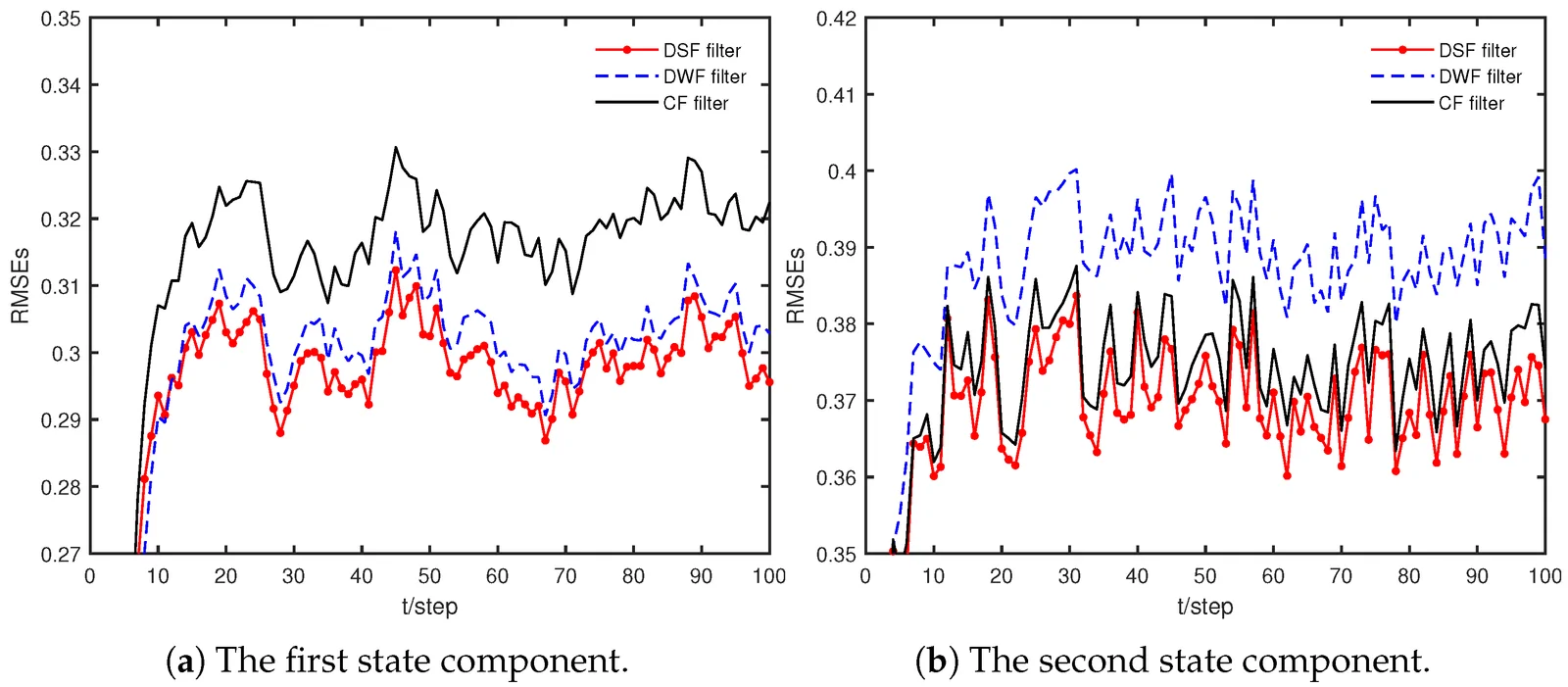
RMSEs of DSF filter, DWF filter in [28], and CF filter in [21] under reception probabilities 0.9, 0.85 and 0.8, respectively.

**Figure 7:**
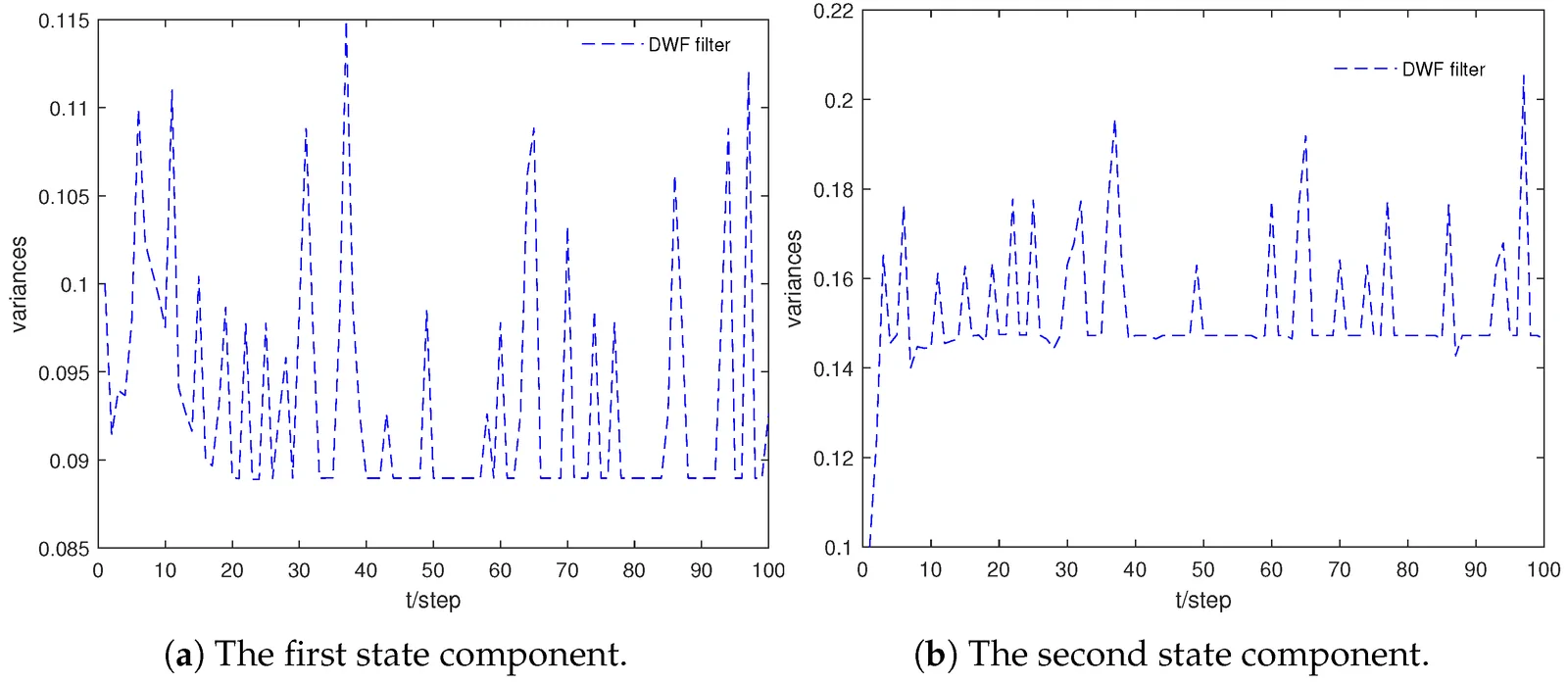
Estimation error variances of DWF filter in [28].

Under reception probabilities $\alpha_{1}=0.9$, $\alpha_{2}=0.85$ and $\alpha_{3}=0.8$, Figure 6 shows the RMSE curves of the three fusion filters (DSF filter, CF filter, DWF filter). The RMSE of the proposed DSF filter is lower than that of the other two compared filters. Figure 7 shows the estimation variance for the DWF filter in [28]. As illustrated in Figure 7, the DWF filter cannot achieve steady-state behavior, as it depends on the random variables $\gamma_{i}\left(t\right)$, i=1,2,3.

**Figure 8:**
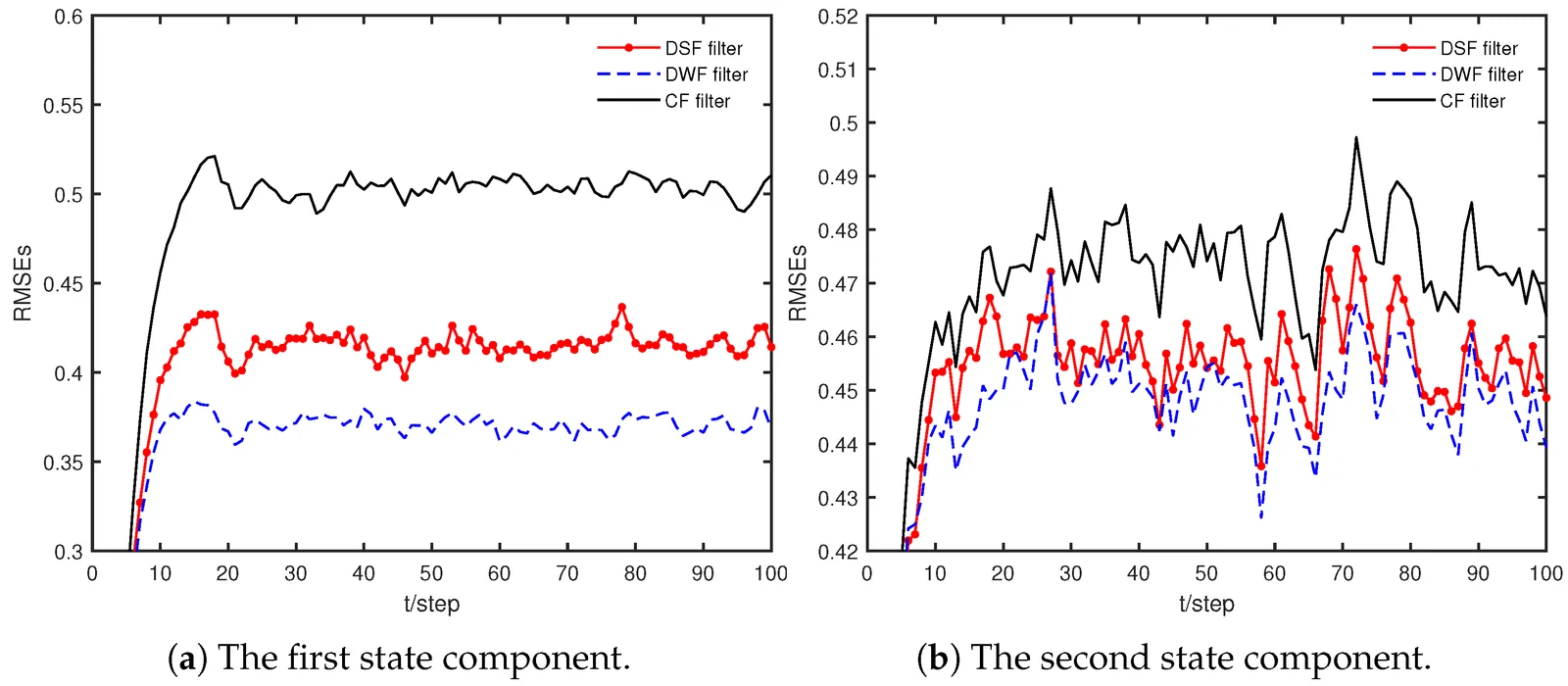
RMSEs of DSF filter, DWF filter in [28], and CF filter in [21] under reception probabilities 0.3, 0.5 and 0.4.

For further comparison, we set $\alpha_{1}=0.3$, $\alpha_{2}=0.5$ and $\alpha_{3}=0.4$, i.e., under low reception probabilities. Figure 8 shows the RMSE for the DSF filter, the CF filter in [21] and the DWF filter in [28]. Specifically, the CF filter exhibits the highest RMSE; the DSF filter achieves a medium level with improved accuracy over the CF filter; and the DWF filter maintains the lowest RMSE throughout, since the DWF filter depends on random variables. However, the DWF filter is only applicable to the case of independent noises, and it does not have steady-state behavior.

Table 5 shows the ARMSE values and percentage improvements of the proposed DSF filter over the CF [21] and DWF filter [28] under both high and low reception probabilities with uncorrelated noise. As shown in Table 5, under high reception probabilities, the DSF filter outperforms both the CF filter and the DWF filter, whereas under low reception probabilities, the DWF filter achieves lower ARMSEs than the DSF filter (indicated by the negative improvements), which is consistent with Figure 8.

### 6.2. Example 2

In this example, the steady-state case is considered. It shows the existence of the steady-state filter when Assumptions 3 and 4 are satisfied. Unlike Cases 1 and 2, where the system parameters do not satisfy these assumptions, the system matrices in this example are modified as follows. All other variables remain the same as in Case 1.

$$x\left(t+1\right)=\left[\begin{array}{cc} 0.9 & T\\ 0 & 0.9 \end{array}\right]x\left(t\right)+\left[\begin{array}{c} T^{2}/2\\ T \end{array}\right]w\left(t\right).$$

In this case, $\mathrm{rank}\left[\begin{array}{c} H_{1}\\ H_{1}\Phi \end{array}\right]=2$, $\mathrm{rank}\left[\begin{array}{c} H_{2}\\ H_{2}\Phi \end{array}\right]=2$, $\mathrm{rank}\left[\begin{array}{c} H_{3}\\ H_{3}\Phi \end{array}\right]=2$ and $\mathrm{rank}\left[\Gamma ,\Phi \Gamma \right]=2$; i.e., the system is completely controllable and completely observable. It means that $\left(\Phi ,H_{i}\right)$ is detectable, and $\left(\Phi -\Gamma S_{i}{\left(Q_{i}^{v}\right)}^{-1}H_{i},{\bar{R}}_{i}\right)$ is stabilizable; according to Assumption 3, the local estimators have steady-state properties. In this example, $\rho (\hat{\bar{\Phi }}⊗\hat{\bar{\Phi }}+\sum_{i=1}^{l}\alpha_{i}\left(1-\alpha_{i}\right)I_{i}{\hat{\bar{\Phi }}}^{\left(1\right)}⊗{\hat{\bar{\Phi }}}^{\left(1\right)}I_{i})=0.81<1$; i.e., Assumption 4 is also satisfied. From Theorem 3, the steady-state filter exists.

**Figure 9:**
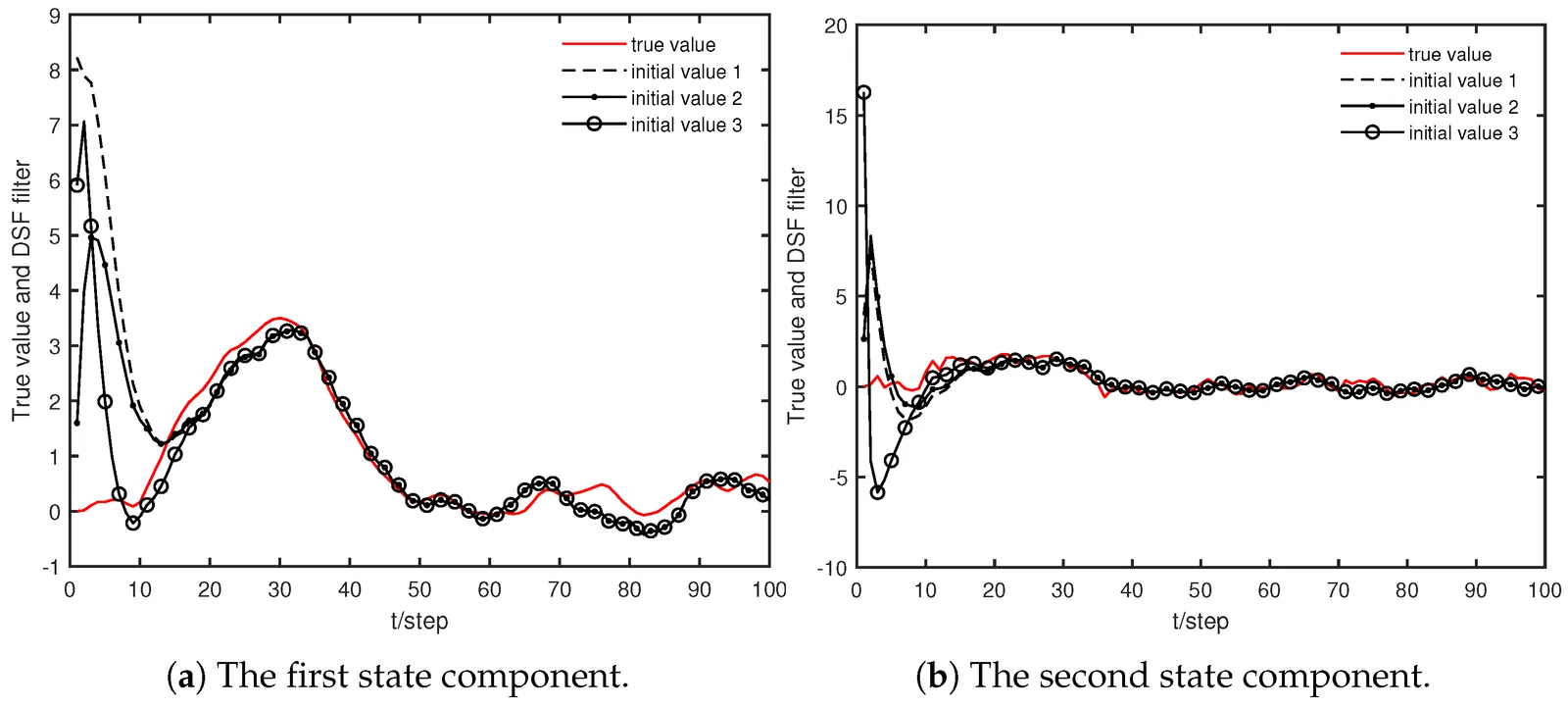
Tracking performance of DSF filter under different initial values.

To illustrate the steady-state behavior of the proposed DSF filter, Figure 9 compares its tracking performance under three different initial values: initial value 1: ${\hat{X}}^{s}\left(0|-1\right)={\left[14\;13\;12\;11\;10\;9\;8\;7\;6\;5\;4\;3\;2\;1\right]}^{T}$ and P(0|−1)=0.1I, initial value 2: ${\hat{X}}^{s}\left(0|-1\right)={\left[1\;2\;3\;4\;5\;6\;7\;8\;9\;10\;11\;12\;13\;14\right]}^{T}$ and P(0|−1)=I, initial value 3: ${\hat{X}}^{s}\left(0|-1\right)={\left[1\;5\;0.5\;3\;10\;0\;7\;-1\;-2\;-3\;-4\;-5\;-6\;-7\right]}^{T}$ and P(0|−1)=5I. The results show that the steady-state DSF filter is asymptotically stable and that the initial values can be chosen arbitrarily.

As shown in Table 6, the estimation errors remain unchanged from the 80th step to the 100th step, which indicates that the DSF filter has reached its steady state. This is consistent with the theoretical result in Theorem 4 and further verifies the steady-state property of the proposed filter.

All simulations were carried out on a Xiaomi laptop equipped with an Intel Core 5 210H CPU (2.20 GHz). The total computational time of the real-time fusion algorithm over the 100-step simulation is 0.031207500 s, whereas that of the steady-state fusion algorithm is only 0.002913800 s. This demonstrates that the steady-state gains pre-computed offline reduce the total computational time, which effectively alleviate the online computational burden of the proposed DSF filter.

## 7. Conclusions

This paper focuses on a distributed fusion filtering problem with prediction-based compensation for DoS-induced packet dropouts modeled as Bernoulli random variables. A distributed fusion filtering algorithm has been developed for multi-sensor systems whose transmission channels from local estimators to the fusion center are subject to random packet dropouts. Such packet dropouts are caused by various random events, in particular, DoS attacks. When a local estimate is lost, a prediction compensation strategy is employed at the fusion center, where the missing data are replaced by their one-step predictors. The DSF filter is derived via the innovation analysis method based on an augmented new system. The proposed framework accommodates correlated process and measurement noises as well as sensors with heterogeneous dropout rates. The steady-state behavior of the proposed DSF filter is rigorously analyzed, and a sufficient condition for the existence of the steady-state filter is established. Under the steady-state condition, the filter gains and error covariances converge to constant matrices. This will reduce the computational and communication burden. And the initial values for the steady-state filter can be chosen arbitrarily. Compared with the existing centralized fusion filter with packet dropouts in [21], the proposed DSF filter achieves superior estimation accuracy under the same reception probabilities. The proposed DSF filter is intermediate in RMSE compared with DWF in [28] under low reception probabilities and independent noise. Under correlated noise conditions, however, DSF is applicable whereas DWF is not.

The current model cannot distinguish DoS attacks from ordinary channel losses. Future work will consider intelligent and time-correlated attack scenarios, design corresponding filter algorithms for time-varying attack probabilities, and explore corresponding attack-detection mechanisms.

## Figures and Tables

**Table 1:** Related work [13,14,15,16,21,25,26,27,28,29,30]: comparison with this paper.

Ref.	Type of Lost Data	Compensation Strategy	Noise-Correlation Assumptions	Steady-State Existence
[21]	measurements	PC	Correlated	Not discussed
[25]	measurements	PC	Uncorrelated	No
[13]	measurements	PC	Partially correlated	Not discussed
[14]	measurements	PC	Unknown but bounded	Not discussed
[15]	measurements	PC	Temporally correlated	Not discussed
[16]	measurements	PC	Correlated	Yes
[26]	measurements	PC	Uncorrelated	Yes
[27]	State estimates	PC	Uncorrelated	Boundedness
[28]	State estimates	PC	Uncorrelated	No
[29]	State estimates	ZI	Uncorrelated	Yes
[30]	State estimates	ZI	Uncorrelated	Boundedness
This paper	State estimates	PC	Correlated	Yes

“Not discussed” means that the corresponding property is not addressed in the reference; “Partially correlated” means that the process noise is uncorrelated with the measurement noises, but the measurement noises of different sensors are correlated with each other; “temporally correlated” means that the measurement noise of each sensor is temporally correlated (modeled as an autoregressive process) but is uncorrelated with all other random variables.

**Table 2:** Dimensions of the variables and matrices in the transformed system (14)–(16).

Symbol	Definition	Dimension
X(t)	Augmented state in (14)	(2l+1)n×1
z(t)	Augmented measurement in (15)	ln×1
$\bar{\Phi }\left(t\right)$	Transition matrix of the augmented system	(2l+1)n×(2l+1)n
H(t)	Measurement matrix of the augmented system	ln×(2l+1)n
D(t)	Coefficient matrix of w(t) in (15)	ln×r
$\Gamma_{0}\left(t\right)$	Coefficient matrix of w(t) in $\bar{w}\left(t\right)$	(2l+1)n×r
$\Gamma_{1}$	Coefficient matrix of w(t+1) in $\bar{w}\left(t\right)$	(2l+1)n×r
$\Gamma_{2}\left(t+1\right)$	Coefficient matrix of v(t+1) in $\bar{w}\left(t\right)$	(2l+1)n×m

Here, $m=\sum_{i=1}^{l}m_{i}$ is the total dimension of the stacked measurement noise v(t).

**Table 3:** ARMSEs and percentage improvements of the proposed DSF filter versus CF [21] in the correlated noise case.

Fusion Filter	DSF Filter	CF Filter in [21]	Improvement
First component	0.2980	0.3205	7.02%
Second component	0.3362	0.3460	2.81%

**Table 4:** Percentage ARMSE increase of the DSF filter under reception probability mismatch.

Reception Probability Mismatch	First Component	Second Component
+30.00% ($\alpha_{1}+0.15$)	0.18%	0.29%
+25.00% ($\alpha_{2}+0.15$)		
+21.42% ($\alpha_{3}+0.15$)		
−30.00% ($\alpha_{1}-0.15$)	1.05%	0.55%
−25.00% ($\alpha_{2}-0.15$)		
−21.42% ($\alpha_{3}-0.15$)		

**Table 5:** ARMSE comparison and percentage improvement of the proposed DSF filter over CF [21] and DWF [28] under high and low reception probabilities.

**Reception** **Probability**	**State** **Component**	**DSF** **(Proposed)**	**CF [21]**	**Improvement**
High	First	0.2991	0.3196	+6.41%
High	Second	0.3718	0.3764	+1.21%
Low	First	0.4197	0.5063	+17.11%
Low	Second	0.4597	0.4790	+4.02%
**Reception** **Probability**	**State** **Component**	**DSF** **(Proposed)**	**DWF [28]**	**Improvement**
High	First	0.2991	0.3042	+1.68%
High	Second	0.3718	0.3905	+4.76%
Low	First	0.4197	0.3742	−12.13%
Low	Second	0.4597	0.4518	−1.74%

**Table 6:** Steady-state filter error variance of the DSF filter at different time steps.

Step	80	90	100
First component	0.04687722996758	0.04687722996758	0.04687722996758
Second component	0.08508771245807	0.08508771245807	0.08508771245807

## Data Availability

The original contributions presented in this study are included in the article. Further inquiries can be directed to the corresponding author.

## Appendix A

To facilitate understanding of the subsequent proofs, cross-references are provided for the main symbols and notations that appear repeatedly in the appendices.

**Table A1:** Summary of the key symbols and notations used in the appendices.

Symbol and Notations	Meaning	Index
$z_{i}\left(t\right)$	Virtual measurement	(6)
X(t)	Augmented state composed of x(t), ${\tilde{x}}_{i}\left(t+1|t\right)$ and $z_{i}\left(t-1\right)$	(14)
z(t)	Augmented measurement composed of $z_{i}\left(t\right)$	(15)
$\bar{w}\left(t\right)$	Augmented noise of system (14), one-step autocorrelated	(16)
$\bar{\Phi }\left(t\right)$	Transition matrix of the augmented system	(14)
H(t)	Measurement matrix	(15)
D(t)	Matrix multiplying w(t) in (15)	(15)
$\Gamma_{0}\left(t\right)$, $\Gamma_{1}$, $\Gamma_{2}\left(t\right)$	Coefficient matrices of w(t), w(t+1) and v(t+1) in $\bar{w}\left(t\right)$	(16)
Λ(t)	Matrix such that $\tilde{\bar{\Phi }}\left(t\right)=\Lambda \left(t\right){\hat{\bar{\Phi }}}^{\left(1\right)}$	Remark 5
α	$\mathrm{diag}(\alpha_{1}I,\cdots ,\alpha_{l}I)$	Remark 5
${\hat{\bar{\Phi }}}^{\left(1\right)}$, ${\hat{H}}^{\left(1\right)}$, ${\hat{\Gamma }}_{0}^{\left(1\right)}$	Deterministic matrices in the fluctuation decompositions	Remark 5
*A*	$\mathrm{diag}(\left(\alpha_{1}-\alpha_{1}^{2}\right)1_{nn},\cdots ,\left(\alpha_{l}-\alpha_{l}^{2}\right)1_{nn})$	Lemma 2

The explicit entries of the matrices are provided in the main text, which can be directly retrieved through the index.

**Proof of Lemma 2.** Under Assumption 1, substituting (16) into $Q^{\bar{w}}\left(t\right)=E\left[\bar{w}\left(t\right){\bar{w}}^{T}\left(t\right)\right]$ yields

(A1) $$\begin{array}{cc} Q^{\bar{w}}\left(t\right) & =E\left[\Gamma_{0}\left(t\right)w\left(t\right)w^{T}\left(t\right)\Gamma_{0}^{T}\left(t\right)\right]+\Gamma_{1}Q^{w}\Gamma_{1}^{T}+\Gamma_{2}\left(t+1\right)Q^{v}\Gamma_{2}^{T}\left(t+1\right)\\ \; & \;+\Gamma_{1}S\Gamma_{2}^{T}\left(t+1\right)+\Gamma_{2}\left(t+1\right)S^{T}\Gamma_{1}^{T}, \end{array}$$

where the property that $\gamma_{i}\left(t\right),i=1,2,\cdots ,l$ are uncorrelated with other variables is utilized. Substituting ${\tilde{\Gamma }}_{0}\left(t\right)=\Gamma_{0}\left(t\right)-{\hat{\Gamma }}_{0}=\Lambda \left(t\right){\hat{\Gamma }}_{0}^{\left(1\right)}$ into $E[\Gamma_{0}\left(t\right)w\left(t\right)w^{T}\left(t\right)\Gamma_{0}^{T}\left(t\right)]$ and using $E[{\tilde{\Gamma }}_{0}\left(t\right)]=0$, we obtain

(A2) $$E\left[\Gamma_{0}\left(t\right)w\left(t\right)w^{T}\left(t\right)\Gamma_{0}^{T}\left(t\right)\right]={\hat{\Gamma }}_{0}Q^{w}{\hat{\Gamma }}_{0}^{T}+E\left\{\Lambda \left(t\right){\hat{\Gamma }}_{0}^{\left(1\right)}w\left(t\right)w^{T}\left(t\right){\left[{\hat{\Gamma }}_{0}^{\left(1\right)}\right]}^{T}\Lambda \left(t\right)\right\}.$$

Using (17), and then from (A2), we obtain

(A3) $$E\left[\Gamma_{0}\left(t\right)w\left(t\right)w^{T}\left(t\right)\Gamma_{0}^{T}\left(t\right)\right]=\left[\begin{array}{cc} 0 & 0\\ 0 & A \end{array}\right]⊙\left\{{\hat{\Gamma }}_{0}^{\left(1\right)}Q^{w}{\left[{\hat{\Gamma }}_{0}^{\left(1\right)}\right]}^{T}\right\}+{\hat{\Gamma }}_{0}Q^{w}{\hat{\Gamma }}_{0}^{T}.$$

Substituting (A3) into (A1) yields (18). Under Assumption 1, substituting (16) into $Q^{\bar{w}w}\left(t\right)=E\left[\bar{w}\left(t\right)w^{T}\left(t\right)\right]$ yields (19). The proof is completed. □

## Appendix B

**Proof of Theorem 1.** Using $\bar{\Phi }\left(t\right)=\hat{\bar{\Phi }}\left(t\right)+\tilde{\bar{\Phi }}\left(t\right)$, system (14) can be rewritten as

(A4) $$X\left(t+1\right)=\hat{\bar{\Phi }}\left(t\right)X\left(t\right)+\tilde{\bar{\Phi }}\left(t\right)X\left(t\right)+\bar{w}\left(t\right),$$

substituting (A4) into $q\left(t+1\right)=E[X\left(t+1\right)X^{T}\left(t+1\right)]$, using $E[\tilde{\bar{\Phi }}\left(t\right)]=0$ and γ(t) being uncorrelated with all other variables, we have

(A5) $$\begin{array}{cc} q(t+1) & =\hat{\bar{\Phi }}\left(t\right)q\left(t\right){\hat{\bar{\Phi }}}^{T}\left(t\right)+\hat{\bar{\Phi }}\left(t\right)Q^{X\bar{w}}\left(t\right)+E\left[\tilde{\bar{\Phi }}\left(t\right)X\left(t\right)X^{T}\left(t\right){\tilde{\bar{\Phi }}}^{T}\left(t\right)\right]\\ \; & \;+E\left[\tilde{\bar{\Phi }}\left(t\right)X\left(t\right){\bar{w}}^{T}\left(t\right)\right]+Q^{\bar{w}X}\left(t\right){\hat{\bar{\Phi }}}^{T}\left(t\right)+E\left[\bar{w}\left(t\right)X^{T}\left(t\right){\tilde{\bar{\Phi }}}^{T}\left(t\right)\right]+Q^{\bar{w}}\left(t\right). \end{array}$$

In the following, the derivation of $E[\tilde{\bar{\Phi }}\left(t\right)X\left(t\right)X^{T}\left(t\right){\tilde{\bar{\Phi }}}^{T}\left(t\right)]$ and $E[\tilde{\bar{\Phi }}\left(t\right)X\left(t\right){\bar{w}}^{T}\left(t\right)]$ are presented. Substituting $\tilde{\bar{\Phi }}\left(t\right)=\Lambda \left(t\right){\hat{\bar{\Phi }}}^{\left(1\right)}$ into $E[\tilde{\bar{\Phi }}\left(t\right)X\left(t\right)X^{T}\left(t\right){\tilde{\bar{\Phi }}}^{T}\left(t\right)]$, and using (17), we have

(A6) $$E\left[\tilde{\bar{\Phi }}\left(t\right)X\left(t\right)X^{T}\left(t\right){\tilde{\bar{\Phi }}}^{T}\left(t\right)\right]=\left[\begin{array}{cc} 0 & 0\\ 0 & A \end{array}\right]⊙\left\{{\hat{\bar{\Phi }}}^{\left(1\right)}q\left(t\right){\left[{\hat{\bar{\Phi }}}^{\left(1\right)}\right]}^{T}\right\}.$$

Substituting $\tilde{\bar{\Phi }}\left(t\right)=\Lambda \left(t\right){\hat{\bar{\Phi }}}^{\left(1\right)}$, ${\tilde{\Gamma }}_{0}\left(t\right)=\Gamma_{0}\left(t\right)-{\hat{\Gamma }}_{0}=\Lambda \left(t\right){\hat{\Gamma }}_{0}^{\left(1\right)}$ into $E[\tilde{\bar{\Phi }}\left(t\right)X\left(t\right){\bar{w}}^{T}\left(t\right)]$ and using $E[\tilde{\bar{\Phi }}\left(t\right)]=0$, $E[{\tilde{\Gamma }}_{0}\left(t\right)]=0$, the property that variable γ(t) is uncorrelated with all other variables, it can be obtained from (17)

(A7) $$E\left[\tilde{\bar{\Phi }}\left(t\right)X\left(t\right){\bar{w}}^{T}\left(t\right)\right]=\left[\begin{array}{cc} 0 & 0\\ 0 & A \end{array}\right]⊙\left\{{\hat{\bar{\Phi }}}^{\left(1\right)}Q^{Xw}\left(t\right){\left[{\hat{\Gamma }}_{0}^{\left(1\right)}\right]}^{T}\right\}.$$

Substituting (A6) and (A7) into (A5), after rearrangement, we can obtain (20). Finally, we present the proof of the initial values q(0). Substituting $z_{i}\left(-1\right)={\hat{x}}_{i}\left(0|-1\right)$, i=1,2,⋯,l into X(0) yields $X\left(0\right)=\left[x^{T}\left(0\right)\;{\tilde{x}}_{1}^{T}\left(1|0\right)\;\cdots \;{\tilde{x}}_{l}^{T}\left(1|0\right)\;{\hat{x}}_{1}^{T}\left(0|-1\right)\;\cdots \right.{\left.{\hat{x}}_{l}^{T}\left(0|-1\right)\right]}^{T}$, substituting it into $q\left(0\right)=E[X\left(0\right)X^{T}\left(0\right)]$ yields

(A8) $$q\left(0\right)=\left[\begin{array}{ccc} E\{x\left(0\right)x^{T}\left(0\right)\} & q^{x\tilde{x}}\left(0\right) & q^{x\hat{x}}\left(0\right)\\ q^{\tilde{x}x}\left(0\right) & q^{\tilde{x}\tilde{x}}\left(0\right) & q^{\tilde{x}\hat{x}}\left(0\right)\\ q^{\hat{x}x}\left(0\right) & q^{\hat{x}\tilde{x}}\left(0\right) & q^{\hat{x}\hat{x}}\left(0\right) \end{array}\right],$$

where $q^{x\tilde{x}}\left(0\right)=\left(E\left\{x\left(0\right){\tilde{x}}_{i}^{T}\left(1|0\right)\right\}\right)$ and $q^{x\hat{x}}\left(0\right)=\left(E\left\{x\left(0\right){\hat{x}}_{i}^{T}\left(0|-1\right)\right\}\right)$, i=1,2,⋯,l, are n×nl matrices, $q^{\tilde{x}\tilde{x}}\left(0\right)=\left(E\left\{{\tilde{x}}_{i}\left(1|0\right){\tilde{x}}_{j}^{T}\left(1|0\right)\right\}\right)$, $q^{\tilde{x}\hat{x}}\left(0\right)=\left(E\left\{{\tilde{x}}_{i}\left(1|0\right){\hat{x}}_{j}^{T}\left(0|-1\right)\right\}\right)$ and $q^{\hat{x}\hat{x}}\left(0\right)=E\left\{{\hat{x}}_{i}\left(0|-1\right){\hat{x}}_{j}^{T}\left(0|-1\right)\right\}$, i,j=1,2,⋯,l are nl×nl matrices, and $q^{\tilde{x}x}\left(0\right)={\left[q^{x\tilde{x}}\left(0\right)\right]}^{T}$, $q^{\hat{x}x}\left(0\right)={\left[q^{x\hat{x}}\left(0\right)\right]}^{T}$, $q^{\hat{x}\tilde{x}}\left(0\right)={\left[q^{\tilde{x}\hat{x}}\left(0\right)\right]}^{T}$. From ${\hat{x}}_{i}\left(0|-1\right)⊥{\tilde{x}}_{i}\left(0|-1\right)$, substituting $x\left(0\right)={\hat{x}}_{i}\left(0|-1\right)+{\tilde{x}}_{i}\left(0|-1\right)$ into $E\{x\left(0\right)x^{T}\left(0\right)\}$, it follows from Lemma 1 that $E\left\{x\left(0\right)x^{T}\left(0\right)\right\}=x_{0}x_{0}^{T}+P_{0}$. Similarly, based on ${\hat{x}}_{i}\left(0|-1\right)⊥{\tilde{x}}_{i}\left(0|-1\right)$, $L_{i}\left(0\right)=0$, (13), Assumption 1, Assumption 2 and Lemma 1, we have $E\left\{x\left(0\right){\tilde{x}}_{i}^{T}\left(1|0\right)\right\}=P_{0}\Phi^{T}$, $E\left\{x\left(0\right){\hat{x}}_{i}^{T}\left(0|-1\right)\right\}=x_{0}x_{0}^{T}$, $E\left\{{\tilde{x}}_{i}\left(1|0\right){\tilde{x}}_{j}^{T}\left(1|0\right)\right\}=\Phi P_{0}\Phi^{T}+\Gamma Q^{w}\Gamma^{T}$, $E\{{\tilde{x}}_{i}\left(1|0\right){\hat{x}}_{j}^{T}\left(0|-1\right)\}=0$, $E\left\{{\hat{x}}_{i}\left(0|-1\right){\hat{x}}_{j}^{T}\left(0|-1\right)\right\}=x_{0}x_{0}^{T}$, where the unbiasedness of the projection $E[{\tilde{x}}_{i}\left(1|0\right)]=0$ is used. The proof is completed. □

## Appendix C

**Proof of Theorem 2.** From the recursive projection formula [33], we directly obtain (21), where the filter gain matrix $K\left(t\right)=E\left[X\left(t\right)\varepsilon^{T}\left(t\right)\right]{\left\{E\left[\varepsilon \left(t\right)\varepsilon^{T}\left(t\right)\right]\right\}}^{-1}$. According to Remark 2, we have w(t+1)⊥L(z(1),z(2),⋯,z(t)), v(t+1)⊥L(z(1),z(2),⋯,z(t)), w(t)⊥L(z(1),z(2),⋯,z(t−1)); thus, based on (14), (22) is obtained similarly with (21), where the predictor gain matrix $L\left(t\right)=E\left\{\left[\bar{\Phi }\left(t\right)X\left(t\right)+\Gamma_{0}\left(t\right)w\left(t\right)\right]\varepsilon^{T}\left(t\right)\right\}{\left\{E\left[\varepsilon \left(t\right)\varepsilon^{T}\left(t\right)\right]\right\}}^{-1}$. Substituting (15) into ε(t)=z(t)−proj[z(t)∣z(1),z(2),⋯,z(t−1)], (23) is obtained, where w(t)⊥L(z(1),z(2),⋯,z(t−1)) is used. Substituting $H\left(t\right)=\hat{H}+\tilde{H}\left(t\right)$, $D\left(t\right)=\hat{D}+\tilde{D}\left(t\right)$ into (15), (23) can be rewritten as

(A9) $$\varepsilon \left(t\right)=\hat{H}\tilde{X}\left(t|t-1\right)+\tilde{H}\left(t\right)X\left(t\right)+\hat{D}w\left(t\right)+\tilde{D}\left(t\right)w\left(t\right),$$

where the one-step prediction error is defined as $\tilde{X}\left(t|t-1\right)=X\left(t\right)-\hat{X}\left(t|t-1\right)$. Based on $E[\tilde{H}\left(t\right)]=0$ and $E[\tilde{D}\left(t\right)]=0$, substituting (A9) into innovation covariance matrix $Q^{\varepsilon }\left(t\right)=E\left[\varepsilon \left(t\right)\varepsilon^{T}\left(t\right)\right]$, we have

(A10) $$\begin{array}{cc} Q^{\varepsilon }\left(t\right) & =\hat{H}P\left(t|t-1\right){\hat{H}}^{T}+\hat{H}E\left[\tilde{X}\left(t|t-1\right)w^{T}\left(t\right)\right]{\hat{D}}^{T}+E\left[\tilde{H}\left(t\right)X\left(t\right)X^{T}\left(t\right){\tilde{H}}^{T}\left(t\right)\right]\\ & \;+E\left[\tilde{H}\left(t\right)X\left(t\right)w^{T}\left(t\right){\tilde{D}}^{T}\left(t\right)\right]+\hat{D}E\left[w\left(t\right){\tilde{X}}^{T}\left(t|t-1\right)\right]{\hat{H}}^{T}+\hat{D}Q^{w}{\hat{D}}^{T}\\ \; & \;+E\left[\tilde{D}\left(t\right)w\left(t\right)X^{T}\left(t\right){\tilde{H}}^{T}\left(t\right)\right]+E\left[\tilde{D}\left(t\right)w\left(t\right)w^{T}\left(t\right){\tilde{D}}^{T}\left(t\right)\right], \end{array}$$

where the prediction error covariance matrix $P\left(t|t-1\right)=E[\tilde{X}\left(t|t-1\right){\tilde{X}}^{T}\left(t|t-1\right)]$. From the projection theory, we have $\hat{X}\left(t|t-1\right)\in L\left(z\left(1\right),z\left(2\right),\cdots ,z\left(t-1\right)\right)$, but w(t)⊥L(z(1),z(2),⋯,z(t−1)); thus, we have $w\left(t\right)⊥\hat{X}\left(t|t-1\right)$, and based on $\tilde{X}\left(t|t-1\right)=X\left(t\right)-\hat{X}\left(t|t-1\right)$, we have

(A11) $$E\left[\tilde{X}\left(t|t-1\right)w^{T}\left(t\right)\right]=E\left[X\left(t\right)w^{T}\left(t\right)\right]=Q^{Xw}\left(t\right).$$

Using $\tilde{H}\left(t\right)=\left[\gamma \left(t\right)-\alpha \right]{\hat{H}}^{\left(1\right)}$ and $\tilde{D}\left(t\right)=\left[\gamma \left(t\right)-\alpha \right]\Gamma^{\left(1\right)}$, under Remark 4 and (17), we have, respectively,

(A12) $$E\left[\tilde{H}\left(t\right)X\left(t\right)X^{T}\left(t\right){\tilde{H}}^{T}\left(t\right)\right]=A⊙\left\{{\hat{H}}^{\left(1\right)}q\left(t\right){\left[{\hat{H}}^{\left(1\right)}\right]}^{T}\right\},$$

(A13) $$E\left[\tilde{H}\left(t\right)X\left(t\right)w^{T}\left(t\right){\tilde{D}}^{T}\left(t\right)\right]=A⊙\left\{{\hat{H}}^{\left(1\right)}Q^{Xw}\left(t\right){\left[\Gamma^{\left(1\right)}\right]}^{T}\right\},$$

(A14) $$E\left[\tilde{D}\left(t\right)w\left(t\right)w^{T}\left(t\right){\tilde{D}}^{T}\left(t\right)\right]=A⊙\left\{\Gamma^{\left(1\right)}Q^{w}{\left[\Gamma^{\left(1\right)}\right]}^{T}\right\},$$

substituting (A11)–(A14) into (A10), after rearrangement, (24) is obtained. In the following, we derive the filter gain matrix K(t) and the prediction gain matrix L(t). Substituting (A9) into $E[X\left(t\right)\varepsilon^{T}\left(t\right)]$, using $E[\tilde{H}\left(t\right)]=0$ and $E[\tilde{D}\left(t\right)]=0$, we have

(A15) $$E\left[X\left(t\right)\varepsilon^{T}\left(t\right)\right]=E\left[X\left(t\right){\tilde{X}}^{T}\left(t|t-1\right)\right]{\hat{H}}^{T}+Q^{Xw}\left(t\right){\hat{D}}^{T}.$$

Based on $\tilde{X}\left(t|t-1\right)=X\left(t\right)-\hat{X}\left(t|t-1\right)$ and $\hat{X}\left(t|t-1\right)⊥\tilde{X}\left(t|t-1\right)$, (A15) can be written as $E\left[X\left(t\right)\varepsilon^{T}\left(t\right)\right]=P\left(t|t-1\right){\hat{H}}^{T}+Q^{Xw}\left(t\right){\hat{D}}^{T}$; thus, (25) can be obtained. Substituting (A9), $\bar{\Phi }\left(t\right)=\tilde{\bar{\Phi }}\left(t\right)+\hat{\bar{\Phi }}\left(t\right)$ and $\Gamma_{0}\left(t\right)={\tilde{\Gamma }}_{0}\left(t\right)+{\hat{\Gamma }}_{0}$ into $E\{\left[\bar{\Phi }\left(t\right)X\left(t\right)+\Gamma_{0}\left(t\right)w\left(t\right)\right]\varepsilon^{T}\left(t\right)\}$, based on $E[\tilde{\bar{\Phi }}\left(t\right)]=0$ and $E[{\tilde{\Gamma }}_{0}\left(t\right)]=0$, we have

(A16) $$\begin{array}{cc} & E\{\left[\bar{\Phi }\left(t\right)X\left(t\right)+\Gamma_{0}\left(t\right)w\left(t\right)\right]\varepsilon^{T}\left(t\right)\}\\ & \;=E\left[\tilde{\bar{\Phi }}\left(t\right)X\left(t\right)X^{T}\left(t\right){\tilde{H}}^{T}\left(t\right)\right]+E\left[\tilde{\bar{\Phi }}\left(t\right)X\left(t\right)w^{T}\left(t\right){\tilde{D}}^{T}\left(t\right)\right]\\ & \;+\hat{\bar{\Phi }}\left(t\right)E\left[X\left(t\right){\tilde{X}}^{T}\left(t|t-1\right)\right]{\hat{H}}^{T}+\hat{\bar{\Phi }}\left(t\right)Q^{Xw}\left(t\right){\hat{D}}^{T}+E\left[{\tilde{\Gamma }}_{0}\left(t\right)w\left(t\right)X^{T}\left(t\right){\tilde{H}}^{T}\left(t\right)\right]\\ \; & \;+E\left[{\tilde{\Gamma }}_{0}\left(t\right)w\left(t\right)w^{T}\left(t\right){\tilde{D}}^{T}\left(t\right)\right]+{\hat{\Gamma }}_{0}E\left[w\left(t\right){\tilde{X}}^{T}\left(t|t-1\right)\right]{\hat{H}}^{T}+{\hat{\Gamma }}_{0}Q^{w}{\hat{D}}^{T}. \end{array}$$

Based on $\tilde{\bar{\Phi }}\left(t\right)=\Lambda \left(t\right){\hat{\bar{\Phi }}}^{\left(1\right)}$, ${\tilde{\Gamma }}_{0}\left(t\right)=\Lambda \left(t\right){\hat{\Gamma }}_{0}^{\left(1\right)}$, $\tilde{D}\left(t\right)=\left[\gamma \left(t\right)-\alpha \right]\Gamma^{\left(1\right)}$, and $\tilde{H}\left(t\right)=\left[\gamma \left(t\right)-\alpha \right]{\hat{H}}^{\left(1\right)}$, from (17), we have, respectively,

(A17) $$E\left[\tilde{\bar{\Phi }}\left(t\right)X\left(t\right)X^{T}\left(t\right){\tilde{H}}^{T}\left(t\right)\right]=\left[\begin{array}{c} 0\\ A \end{array}\right]⊙\left\{{\hat{\bar{\Phi }}}^{\left(1\right)}q\left(t\right){\left[{\hat{H}}^{\left(1\right)}\right]}^{T}\right\},$$

(A18) $$E\left[\tilde{\bar{\Phi }}\left(t\right)X\left(t\right)w^{T}\left(t\right){\tilde{D}}^{T}\left(t\right)\right]=\left[\begin{array}{c} 0\\ A \end{array}\right]⊙\left\{{\hat{\bar{\Phi }}}^{\left(1\right)}Q^{Xw}\left(t\right){\left[\Gamma^{\left(1\right)}\right]}^{T}\right\},$$

(A19) $$E\left[{\tilde{\Gamma }}_{0}\left(t\right)w\left(t\right)X^{T}\left(t\right){\tilde{H}}^{T}\left(t\right)\right]=\left[\begin{array}{c} 0\\ A \end{array}\right]⊙\left\{{\hat{\Gamma }}_{0}^{\left(1\right)}Q^{wX}\left(t\right){\left[{\hat{H}}^{\left(1\right)}\right]}^{T}\right\},$$

(A20) $$E\left[{\tilde{\Gamma }}_{0}\left(t\right)w\left(t\right)w^{T}\left(t\right){\tilde{D}}^{T}\left(t\right)\right]=\left[\begin{array}{c} 0\\ A \end{array}\right]⊙\left\{{\hat{\Gamma }}_{0}^{\left(1\right)}Q^{w}{\left[\Gamma^{\left(1\right)}\right]}^{T}\right\}.$$

In the derivation of (A11), $w\left(t\right)⊥\hat{X}\left(t|t-1\right)$ is used, and based on $\tilde{X}\left(t|t-1\right)=X\left(t\right)-\hat{X}\left(t|t-1\right)$, we have, respectively,

(A21) $$\hat{\bar{\Phi }}\left(t\right)E\left[X\left(t\right){\tilde{X}}^{T}\left(t|t-1\right)\right]{\hat{H}}^{T}=\hat{\bar{\Phi }}\left(t\right)P\left(t|t-1\right){\hat{H}}^{T},$$

(A22) $${\hat{\Gamma }}_{0}E\left[w\left(t\right){\tilde{X}}^{T}\left(t|t-1\right)\right]{\hat{H}}^{T}={\hat{\Gamma }}_{0}Q^{wX}\left(t\right){\hat{H}}^{T}.$$

Substituting (A16)–(A22) into $L\left(t\right)=E\left\{\left[\bar{\Phi }\left(t\right)X\left(t\right)+\Gamma_{0}\left(t\right)w\left(t\right)\right]\varepsilon^{T}\left(t\right)\right\}{\left\{E\left[\varepsilon \left(t\right)\varepsilon^{T}\left(t\right)\right]\right\}}^{-1}$, (26) can be obtained. Subtracting (21) from state X(t) yields the filtering error $\tilde{X}\left(t|t\right)=\tilde{X}\left(t|t-1\right)-K\left(t\right)\varepsilon \left(t\right)$; by rearranging the terms, we have

(A23) $$\tilde{X}\left(t|t\right)+K\left(t\right)\varepsilon \left(t\right)=\tilde{X}\left(t|t-1\right).$$

It follows from $\tilde{X}\left(t|t\right)⊥\varepsilon \left(t\right)$, taking the variance on both sides of (A23), we have $P\left(t|t\right)+K\left(t\right)Q^{\varepsilon }\left(t\right)K^{T}\left(t\right)=P\left(t|t-1\right)$ where $P\left(t|t\right)=E[\tilde{X}\left(t|t\right){\tilde{X}}^{T}\left(t|t\right)]$, $P\left(t|t-1\right)=E[\tilde{X}\left(t|t-1\right){\tilde{X}}^{T}\left(t|t-1\right)]$; thus, (27) is obtained. Similarly, subtracting (22) from X(t+1), based on (14), we have $\tilde{X}\left(t+1|t\right)+L\left(t\right)\varepsilon \left(t\right)=\hat{\bar{\Phi }}\left(t\right)\tilde{X}\left(t|t-1\right)+\tilde{\bar{\Phi }}\left(t\right)X\left(t\right)+\bar{w}\left(t\right)$, and taking the variance on both sides, it can be obtained that

(A24) $$\begin{array}{cc} & P\left(t+1|t\right)+L\left(t\right)Q^{\varepsilon }\left(t\right)L^{T}\left(t\right)=E\{\left[\hat{\bar{\Phi }}\left(t\right)\tilde{X}\left(t|t-1\right)+\tilde{\bar{\Phi }}\left(t\right)X\left(t\right)+\bar{w}\left(t\right)\right]\\ \; & \;\;\;\;\;\;\;\;\;\;\;\;\;\;\times {\left[\hat{\bar{\Phi }}\left(t\right)\tilde{X}\left(t|t-1\right)+\tilde{\bar{\Phi }}\left(t\right)X\left(t\right)+\bar{w}\left(t\right)\right]}^{T}\}, \end{array}$$

using $E[\tilde{\bar{\Phi }}\left(t\right)]=0$, the right-hand side of (A24) can be expanded as

(A25) $$\begin{array}{cc} & \hat{\bar{\Phi }}\left(t\right)P\left(t|t-1\right){\hat{\bar{\Phi }}}^{T}\left(t\right)+\hat{\bar{\Phi }}\left(t\right)E\left[\tilde{X}\left(t|t-1\right){\bar{w}}^{T}\left(t\right)\right]+E\left[\tilde{\bar{\Phi }}\left(t\right)X\left(t\right)X^{T}\left(t\right){\tilde{\bar{\Phi }}}^{T}\left(t\right)\right]\\ \; & \;+E\left[\tilde{\bar{\Phi }}\left(t\right)X\left(t\right){\bar{w}}^{T}\left(t\right)\right]+E\left[\bar{w}\left(t\right)\tilde{X}\left(t|t-1\right)\right]{\hat{\bar{\Phi }}}^{T}\left(t\right)+E\left[\bar{w}\left(t\right)X^{T}\left(t\right){\tilde{\bar{\Phi }}}^{T}\left(t\right)\right]+Q^{\bar{w}}\left(t\right). \end{array}$$

It is known that $\hat{X}\left(t|t-1\right)\in L\left(z\left(t-1\right),z\left(t-2\right),\cdots ,z\left(1\right)\right)$, w(t)⊥L(z(t−1),z(t−2),⋯,z(1)), w(t+1)⊥L(z(t−1),z(t−2),⋯,z(1)), v(t+1)⊥L(z(t−1),z(t−2),⋯,z(1)), hence, we have $\bar{w}\left(t\right)⊥\hat{X}\left(t|t-1\right)$. Based on $\tilde{X}\left(t|t-1\right)=X\left(t\right)-\hat{X}\left(t|t-1\right)$ and $\bar{w}\left(t\right)⊥\hat{X}\left(t|t-1\right)$, we have

(A26) $$E\left[\tilde{X}\left(t|t-1\right){\bar{w}}^{T}\left(t\right)\right]=E\left[X\left(t\right){\bar{w}}^{T}\left(t\right)\right]=Q^{X\bar{w}}\left(t\right).$$

Based on $E[\tilde{\bar{\Phi }}\left(t\right)]=0$, $E[{\tilde{\Gamma }}_{0}\left(t\right)]=0$, $\tilde{\bar{\Phi }}\left(t\right)=\Lambda \left(t\right){\hat{\bar{\Phi }}}^{\left(1\right)}$, ${\tilde{\Gamma }}_{0}\left(t\right)=\Lambda \left(t\right){\hat{\Gamma }}_{0}^{\left(1\right)}$, from (17), we have

(A27) $$E\left[\tilde{\bar{\Phi }}\left(t\right)X\left(t\right){\bar{w}}^{T}\left(t\right)\right]=\left[\begin{array}{cc} 0 & 0\\ 0 & A \end{array}\right]⊙\left\{{\hat{\bar{\Phi }}}^{\left(1\right)}Q^{Xw}\left(t\right){\left[{\hat{\Gamma }}_{0}^{\left(1\right)}\right]}^{T}\right\}.$$

Based on $E[\tilde{\bar{\Phi }}\left(t\right)]=0$, $\tilde{\bar{\Phi }}\left(t\right)=\Lambda \left(t\right){\hat{\bar{\Phi }}}^{\left(1\right)}$, from (17), we have

(A28) $$E\left[\tilde{\bar{\Phi }}\left(t\right)X\left(t\right)X^{T}\left(t\right){\tilde{\bar{\Phi }}}^{T}\left(t\right)\right]=\left[\begin{array}{cc} 0 & 0\\ 0 & A \end{array}\right]⊙\left\{{\hat{\bar{\Phi }}}^{\left(1\right)}q\left(t\right){\left[{\hat{\bar{\Phi }}}^{\left(1\right)}\right]}^{T}\right\}.$$

After substituting (A26)–(A28) into (A25), rearranging (A24) yields (28). In the end, we give the proof of the initial values. Substituting $z_{i}\left(-1\right)={\hat{x}}_{i}\left(0|-1\right),i=1,2,\cdots ,l$ into X(0), we have

$$X\left(0\right)={\left[\begin{array}{ccccccc} x^{T}\left(0\right) & {\tilde{x}}_{1}^{T}\left(1|0\right) & \cdots & {\tilde{x}}_{l}^{T}\left(1|0\right) & {\hat{x}}_{1}^{T}\left(0|-1\right) & \cdots & {\hat{x}}_{l}^{T}\left(0|-1\right) \end{array}\right]}^{T}.$$

Under Assumption 2, there is $E\left[x\left(0\right)\right]=x_{0}$; from the unbiasedness of ${\hat{x}}_{i}\left(1|0\right)$, we have $E[{\tilde{x}}_{i}\left(1|0\right)]=0$ and under Lemma 1, there is ${\hat{x}}_{i}\left(0|-1\right)=x_{0},i=1,2,\cdots ,l$. From $E\left[\tilde{X}\left(0|-1\right)\right]=E\left[X\left(0\right)-\hat{X}\left(0|-1\right)\right]=0$, we have $\hat{X}\left(0|-1\right)=E\left[X\left(0\right)\right]={\left[\begin{array}{ccccccc} x_{0}^{T} & 0 & \cdots & 0 & x_{0}^{T} & \cdots & x_{0}^{T} \end{array}\right]}^{T}$. Substituting

$$\tilde{X}\left(0|-1\right)={\left[\begin{array}{ccccccc} {\left(x\left(0\right)-x_{0}\right)}^{T} & {\tilde{x}}_{1}^{T}\left(1|0\right) & \cdots & {\tilde{x}}_{l}^{T}\left(1|0\right) & 0 & \cdots & 0 \end{array}\right]}^{T}$$

into $P\left(0|-1\right)=E[\tilde{X}\left(0|-1\right){\tilde{X}}^{T}\left(0|-1\right)]$, we have

$$P\left(0|-1\right)=\left[\begin{array}{ccc} E\{\left(x\left(0\right)-x_{0}\right){\left(x\left(0\right)-x_{0}\right)}^{T}\} & P^{x\tilde{x}}\left(0|-1\right) & 0\\ P^{\tilde{x}x}\left(0|-1\right) & P^{\tilde{x}\tilde{x}}\left(0|-1\right) & 0\\ 0 & 0 & 0 \end{array}\right],$$

where $P^{x\tilde{x}}\left(0|-1\right)=\left(E\left\{\left(x\left(0\right)-x_{0}\right){\tilde{x}}_{i}^{T}\left(1|0\right)\right\}\right)$ is an n×nl matrix, $P^{\tilde{x}\tilde{x}}\left(0|-1\right)=\left(E\left\{{\tilde{x}}_{i}\left(1|0\right){\tilde{x}}_{j}^{T}\left(1|0\right)\right\}\right)$ is a nl×nl matrix, and $P^{\tilde{x}x}\left(0|-1\right)={\left[P^{x\tilde{x}}\left(0|-1\right)\right]}^{T}$. The derivation of the initial value can be referred to the proof of Theorem 1. The proof is completed. □

## Appendix D

**Proof of Theorem 3.** Under Assumption 3, the local one-step predictor will have the stability [33]. When t→∞, we have $L_{i}\left(t\right)=L_{i}$, $\Psi_{i}\left(t\right)=\Psi_{i}$; furthermore, there will be $L^{a}\left(t\right)=L^{a}$, Ψ(t)=Ψ, $\Gamma_{2}\left(t\right)=\Gamma_{2}$, $\hat{\bar{\Phi }}\left(t\right)=\hat{\bar{\Phi }}$, $Q^{\bar{w}}\left(t\right)=Q^{\bar{w}}$, $Q^{X\bar{w}}\left(t\right)=Q^{X\bar{w}}$, $Q^{Xw}\left(t\right)=Q^{Xw}$. (20) can be rewritten as

(A29) $$q\left(t+1\right)=\hat{\bar{\Phi }}q\left(t\right){\hat{\bar{\Phi }}}^{T}+E\left\{\Lambda \left(t\right){\hat{\bar{\Phi }}}^{\left(1\right)}X\left(t\right)X^{T}\left(t\right){\left[{\hat{\bar{\Phi }}}^{\left(1\right)}\right]}^{T}\Lambda^{T}\left(t\right)\right\}+\Sigma^{q},$$

where $\Sigma^{q}=\hat{\bar{\Phi }}Q^{X\bar{w}}+Q^{\bar{w}X}{\hat{\bar{\Phi }}}^{T}+Q^{\bar{w}}+\left[\begin{array}{cc} 0 & 0\\ 0 & A \end{array}\right]⊙\left\{{\hat{\bar{\Phi }}}^{\left(1\right)}Q^{Xw}{\left[{\hat{\Gamma }}_{0}^{\left(1\right)}\right]}^{T}+{\hat{\Gamma }}_{0}^{\left(1\right)}Q^{wX}{\left[{\hat{\bar{\Phi }}}^{\left(1\right)}\right]}^{T}\right\}$. Based on $\Lambda \left(t\right)=\sum_{i=1}^{l}\left[\gamma_{i}\left(t\right)-\alpha_{i}\right]I_{i}$ and $E[\left(\gamma_{i}\left(t\right)-\alpha_{i}\right)\left(\gamma_{j}\left(t\right)-\alpha_{j}\right)]=0$, we can have

(A30) $$E\left\{\Lambda \left(t\right){\hat{\bar{\Phi }}}^{\left(1\right)}X\left(t\right)X^{T}\left(t\right){\left[{\hat{\bar{\Phi }}}^{\left(1\right)}\right]}^{T}\Lambda^{T}\left(t\right)\right\}=\sum_{i=1}^{l}\alpha_{i}\left(1-\alpha_{i}\right)I_{i}{\hat{\bar{\Phi }}}^{\left(1\right)}q\left(t\right){\left[{\hat{\bar{\Phi }}}^{\left(1\right)}\right]}^{T}I_{i},$$

Substituting (A30) into (A29) yields

(A31) $$q\left(t+1\right)=\hat{\bar{\Phi }}q\left(t\right){\hat{\bar{\Phi }}}^{T}+\sum_{i=1}^{l}\alpha_{i}\left(1-\alpha_{i}\right)I_{i}{\hat{\bar{\Phi }}}^{\left(1\right)}q\left(t\right){\left[{\hat{\bar{\Phi }}}^{\left(1\right)}\right]}^{T}I_{i}+\Sigma^{q},$$

similar to [18], when $\rho (\bar{A})<1$, $\bar{A}=\hat{\bar{\Phi }}⊗\hat{\bar{\Phi }}+\sum_{i=1}^{l}\alpha_{i}\left(1-\alpha_{i}\right)I_{i}{\hat{\bar{\Phi }}}^{\left(1\right)}⊗{\hat{\bar{\Phi }}}^{\left(1\right)}I_{i}$, the solution q(t) to (A31) with any initial value q(0)≥0 converges to the positive semi-definite solution *q* to the algebraic Lyapunov (29), i.e., $q=\lim_{t\to \infty }q\left(t\right)$ finite. The proof is completed. □

## Appendix E

**Proof of Theorem 4.** When t→∞, substituting (A9) into $\tilde{X}\left(t+1|t\right)+L\left(t\right)\varepsilon \left(t\right)=\hat{\bar{\Phi }}\left(t\right)\tilde{X}\left(t|t-1\right)+\tilde{\bar{\Phi }}\left(t\right)X\left(t\right)+\bar{w}\left(t\right)$ and from Theorem 3 yields

(A32) $$\begin{array}{cc} \tilde{X}\left(t+1|t\right)= & \left[\hat{\bar{\Phi }}-L\left(t\right)\hat{H}\right]\tilde{X}\left(t|t-1\right)+\left[\tilde{\bar{\Phi }}\left(t\right)-L\left(t\right)\tilde{H}\left(t\right)\right]X\left(t\right)+\Gamma_{0}\left(t\right)w\left(t\right)\\ \; & +\Gamma_{1}w\left(t+1\right)+\Gamma_{2}v\left(t+1\right)-L\left(t\right)\hat{D}w\left(t\right)-L\left(t\right)\tilde{D}\left(t\right)w\left(t\right). \end{array}$$

Substituting (A32) into $P\left(t+1|t\right)=E[\tilde{X}\left(t+1|t\right){\tilde{X}}^{T}\left(t+1|t\right)]$ and using $E[\tilde{\bar{\Phi }}\left(t\right)]=0$, $E[\tilde{H}\left(t\right)]=0$, $E[{\tilde{\Gamma }}_{0}\left(t\right)]=0$ yield

(A33) $$\begin{array}{cc} \; & P(t+1|t)\\ \; & =E\{\{\left[\hat{\bar{\Phi }}-L\left(t\right)\hat{H}\right]\tilde{X}\left(t|t-1\right)+\left[\tilde{\bar{\Phi }}\left(t\right)-L\left(t\right)\tilde{H}\left(t\right)\right]X\left(t\right)+\Gamma_{0}\left(t\right)w\left(t\right)+\Gamma_{1}w\left(t+1\right)\\ \; & \;+\Gamma_{2}v\left(t+1\right)-L\left(t\right)\hat{D}w\left(t\right)-L\left(t\right)\tilde{D}\left(t\right)w\left(t\right)\}{\left\{*\right\}}^{T}\}\\ \; & =\left[\hat{\bar{\Phi }}-L\left(t\right)\hat{H}\right]P\left(t|t-1\right){\left[\hat{\bar{\Phi }}-L\left(t\right)\hat{H}\right]}^{T}+E\left\{\left[\hat{\bar{\Phi }}-L\left(t\right)\hat{H}\right]\tilde{X}\left(t|t-1\right)w^{T}\left(t\right)\Gamma_{0}^{T}\left(t\right)\right\}\\ \; & \;+E{\left\{*\right\}}^{T}-E\left\{\left[\hat{\bar{\Phi }}-L\left(t\right)\hat{H}\right]\tilde{X}\left(t|t-1\right)w^{T}\left(t\right){\hat{D}}^{T}L^{T}\left(t\right)\right\}-E{\left\{*\right\}}^{T}\\ \; & \;+E\{\left[\tilde{\bar{\Phi }}\left(t\right)-L\left(t\right)\tilde{H}\left(t\right)\right]X\left(t\right)X^{T}\left(t\right){\left[\tilde{\bar{\Phi }}\left(t\right)-L\left(t\right)\tilde{H}\left(t\right)\right]}^{T}\}\\ \; & \;+E\left\{\left[\tilde{\bar{\Phi }}\left(t\right)-L\left(t\right)\tilde{H}\left(t\right)\right]X\left(t\right)w^{T}\left(t\right)\Gamma_{0}^{T}\left(t\right)\right\}+E{\left\{*\right\}}^{T}\\ \; & \;-E\left\{\left[\tilde{\bar{\Phi }}\left(t\right)-L\left(t\right)\tilde{H}\left(t\right)\right]X\left(t\right)w^{T}\left(t\right){\hat{D}}^{T}L^{T}\left(t\right)\right\}-E{\left\{*\right\}}^{T}\\ \; & \;-E\left\{\left[\tilde{\bar{\Phi }}\left(t\right)-L\left(t\right)\tilde{H}\left(t\right)\right]X\left(t\right)w^{T}\left(t\right){\tilde{D}}^{T}\left(t\right)L^{T}\left(t\right)\right\}-E{\left\{*\right\}}^{T}\\ \; & \;+E\left\{\Gamma_{0}\left(t\right)w\left(t\right)w^{T}\left(t\right)\Gamma_{0}^{T}\left(t\right)\right\}+E\left\{\Gamma_{1}w\left(t+1\right)w^{T}\left(t+1\right)\Gamma_{1}^{T}\right\}\\ \; & \;+E\left\{\Gamma_{2}v\left(t+1\right)v^{T}\left(t+1\right)\Gamma_{2}^{T}\right\}+E\left\{\Gamma_{1}w\left(t+1\right)v^{T}\left(t+1\right)\Gamma_{2}^{T}\right\}+E{\left\{*\right\}}^{T}\\ \; & \;-E\left\{\Gamma_{0}\left(t\right)w\left(t\right)w^{T}\left(t\right){\hat{D}}^{T}L^{T}\left(t\right)\right\}-E{\left\{*\right\}}^{T}\\ \; & \;-E\left\{\Gamma_{0}\left(t\right)w\left(t\right)w^{T}\left(t\right){\tilde{D}}^{T}\left(t\right)L^{T}\left(t\right)\right\}-E{\left\{*\right\}}^{T}\\ \; & \;+E\left\{L\left(t\right)\hat{D}w\left(t\right)w^{T}\left(t\right){\hat{D}}^{T}L^{T}\left(t\right)\right\}+E\left\{L\left(t\right)\hat{D}w\left(t\right)w^{T}\left(t\right){\tilde{D}}^{T}\left(t\right)L^{T}\left(t\right)\right\}+E{\left\{*\right\}}^{T}\\ \; & \;+E\{L\left(t\right)\tilde{D}\left(t\right)w\left(t\right)w^{T}\left(t\right){\tilde{D}}^{T}\left(t\right)L^{T}\left(t\right)\}\\ \; & =\left[\hat{\bar{\Phi }}-L\left(t\right)\hat{H}\right]P\left(t|t-1\right){\left[\hat{\bar{\Phi }}-L\left(t\right)\hat{H}\right]}^{T}+\overset{˘}{Q}-L\left(t\right){\overset{˘}{S}}^{T}-\overset{˘}{S}L^{T}\left(t\right)+L\left(t\right)\overset{˘}{R}L^{T}\left(t\right), \end{array}$$

where {∗} denotes the term inside the preceding curly braces, w(t) is uncorrelated with w(t+1) and v(t+1), X(t) is uncorrelated with w(t+1) and v(t+1), $E\left[\tilde{X}\left(t|t-1\right)w^{T}\left(t\right)\right]=Q^{Xw}$. $\overset{˘}{Q}$, $\overset{˘}{S}$, and $\overset{˘}{R}$ are, respectively, defined as follows:

$$\begin{array}{cc} \overset{˘}{Q}= & E\left\{\hat{\bar{\Phi }}\tilde{X}\left(t|t-1\right)w^{T}\left(t\right)\Gamma_{0}^{T}\left(t\right)\right\}+E{\left\{*\right\}}^{T}+E\left\{\tilde{\bar{\Phi }}\left(t\right)X\left(t\right)X^{T}\left(t\right){\tilde{\bar{\Phi }}}^{T}\left(t\right)\right\}+E\{\tilde{\bar{\Phi }}\left(t\right)X\left(t\right)w^{T}\left(t\right)\times \\ \; & \;\Gamma_{0}^{T}{\left(t\right)\}+E\left\{*\right\}}^{T}+E\left\{\Gamma_{0}\left(t\right)w\left(t\right)w^{T}\left(t\right)\Gamma_{0}^{T}\left(t\right)\right\}+E\left\{\Gamma_{1}w\left(t+1\right)w^{T}\left(t+1\right)\Gamma_{1}^{T}\right\}\\ \; & \;+E\left\{\Gamma_{2}v\left(t+1\right)v^{T}\left(t+1\right)\Gamma_{2}^{T}\right\}+E\left\{\Gamma_{1}w\left(t+1\right)v^{T}\left(t+1\right)\Gamma_{2}^{T}\right\}+E{\left\{*\right\}}^{T}\\ = & \hat{\bar{\Phi }}Q^{Xw}{\hat{\Gamma }}_{0}^{T}+{\hat{\Gamma }}_{0}Q^{wX}{\hat{\bar{\Phi }}}^{T}+\Gamma_{1}Q^{w}\Gamma_{1}^{T}+\Gamma_{1}S\Gamma_{2}^{T}+\Gamma_{2}S^{T}\Gamma_{1}^{T}+\Gamma_{2}Q^{v}\Gamma_{2}^{T}\\ \; & +\left[\begin{array}{cc} 0 & 0\\ 0 & A \end{array}\right]⊙\{{\hat{\bar{\Phi }}}^{\left(1\right)}q{\left[{\hat{\bar{\Phi }}}^{\left(1\right)}\right]}^{T}+{\hat{\bar{\Phi }}}^{\left(1\right)}Q^{Xw}{\left[{\hat{\Gamma }}_{0}^{\left(1\right)}\right]}^{T}+{\hat{\Gamma }}_{0}^{\left(1\right)}Q^{w}{\left[{\hat{\Gamma }}_{0}^{\left(1\right)}\right]}^{T}+{\hat{\Gamma }}_{0}^{\left(1\right)}Q^{wX}{\left[{\hat{\bar{\Phi }}}^{\left(1\right)}\right]}^{T}\}, \end{array}$$

$$\begin{array}{cc} \overset{˘}{S}= & E\left\{\Gamma_{0}\left(t\right)w\left(t\right){\tilde{X}}^{T}\left(t|t-1\right){\hat{H}}^{T}\right\}+E\left\{\hat{\bar{\Phi }}\tilde{X}\left(t|t-1\right)w^{T}\left(t\right){\hat{D}}^{T}\right\}+E\left\{\tilde{\bar{\Phi }}\left(t\right)X\left(t\right)X^{T}\left(t\right){\tilde{H}}^{T}\left(t\right)\right\}\\ \; & +E\left\{\Gamma_{0}\left(t\right)w\left(t\right)X^{T}\left(t\right){\tilde{H}}^{T}\left(t\right)\right\}+E\left\{\tilde{\bar{\Phi }}\left(t\right)X\left(t\right)w^{T}\left(t\right){\tilde{D}}^{T}\left(t\right)\right\}+E\left\{\Gamma_{0}\left(t\right)w\left(t\right)w^{T}\left(t\right){\hat{D}}^{T}\right\}\\ \; & +E\{\Gamma_{0}\left(t\right)w\left(t\right)w^{T}\left(t\right){\tilde{D}}^{T}\left(t\right)\}\\ = & \left[\begin{array}{c} 0\\ A \end{array}\right]⊙\{{\hat{\bar{\Phi }}}^{\left(1\right)}q{\left[{\hat{H}}^{\left(1\right)}\right]}^{T}+{\hat{\bar{\Phi }}}^{\left(1\right)}Q^{Xw}{\left[\Gamma^{\left(1\right)}\right]}^{T}+{\hat{\Gamma }}_{0}^{\left(1\right)}Q^{wX}{\left[{\hat{H}}^{\left(1\right)}\right]}^{T}+{\hat{\Gamma }}_{0}^{\left(1\right)}Q^{w}{\left[\Gamma^{\left(1\right)}\right]}^{T}\}\\ \; & +\hat{\bar{\Phi }}Q^{Xw}{\hat{D}}^{T}+{\hat{\Gamma }}_{0}Q^{wX}{\hat{H}}^{T}+{\hat{\Gamma }}_{0}Q^{w}{\hat{D}}^{T}, \end{array}$$

$$\begin{array}{cc} \overset{˘}{R}= & E\left\{\hat{H}\tilde{X}\left(t|t-1\right)w^{T}\left(t\right){\hat{D}}^{T}\right\}+E{\left\{*\right\}}^{T}+E\left\{\tilde{H}\left(t\right)X\left(t\right)X^{T}\left(t\right){\tilde{H}}^{T}\left(t\right)\right\}+E\{\tilde{H}\left(t\right)X\left(t\right)w^{T}\left(t\right)\times \\ \; & {\tilde{D}}^{T}{\left(t\right)\}+E\left\{*\right\}}^{T}+E\left\{\hat{D}w\left(t\right)w^{T}\left(t\right){\hat{D}}^{T}\right\}+E\left\{\tilde{D}\left(t\right)w\left(t\right)w^{T}\left(t\right){\tilde{D}}^{T}\left(t\right)\right\}\\ = & \hat{H}Q^{Xw}{\hat{D}}^{T}+\hat{D}Q^{wX}{\hat{H}}^{T}+\hat{D}Q^{w}{\hat{D}}^{T}\\ \; & +A⊙\{{\hat{H}}^{\left(1\right)}q{\left[{\hat{H}}^{\left(1\right)}\right]}^{T}+{\hat{H}}^{\left(1\right)}Q^{Xw}{\left[\Gamma^{\left(1\right)}\right]}^{T}+\Gamma^{\left(1\right)}Q^{wX}{\left[{\hat{H}}^{\left(1\right)}\right]}^{T}+\Gamma^{\left(1\right)}Q^{w}{\left[\Gamma^{\left(1\right)}\right]}^{T}\}. \end{array}$$

Similar to [18], the solution P(t+1|t) to (28) with any initial condition P(0|−1)≥0 converges to the unique positive semi-definite solution Σ to (30), i.e., $\lim_{t\to \infty }P\left(t|t-1\right)=\Sigma$. Furthermore, $\hat{\bar{\Phi }}-L\hat{H}$ is a stable matrix, which implies the asymptotical stability of the steady-state fusion predictor. The proof is completed. □
